# From a nanoparticular solid-state material to molecular organo-f-element-polyarsenides†‡

**DOI:** 10.1039/d1sc05797a

**Published:** 2022-02-04

**Authors:** Niklas Reinfandt, Adrian Hauser, Luca Münzfeld, Peter W. Roesky

**Affiliations:** Institute of Inorganic Chemistry, Karlsruhe Institute of Technology (KIT) Engesserstr. 15 D-76131 Karlsruhe Germany roesky@kit.edu

## Abstract

A convenient pathway to new molecular organo-lanthanide-polyarsenides in general and to a f-element complex with the largest polyarsenide ligand in detail is reported. For this purpose, the activation of the solid state material As^0^_nano_ (nanoscale gray arsenic) by the multi electron reducing agents [K(18-crown-6)][(
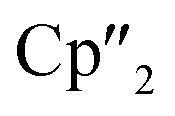
Ln^+II^)_2_(μ-η^6^:η^6^-C_6_H_6_)] (Ln = La, Ce, Cp′′ = 1,3-bis(trimethylsilyl)cyclopentadienyl anion) and [K(18-crown-6)]_2_[(
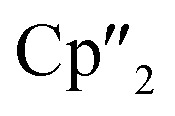
Ln^+II^)_2_(μ-η^6^:η^6^-C_6_H_6_)] (Ln = Ce, Nd) is shown. These non-classical divalent lanthanide compounds were used as three and four electron reducing agents where the product formation can be directed by variation of the applied reactant. The obtained Zintl anions As_3_^3−^, As_7_^3−^, and As_14_^4−^ were previously not accessible in molecular 4f-element chemistry. Additionally, the corresponding compounds with As_14_^4−^-moieties represent the largest organo-lanthanide-polyarsenides known to date.

## Abstract 2

Reaction of non-classical divalent lanthanide compounds with nanoparticulate gray arsenic *via* three- and four-electron reduction led to a series of new f-element polyarsenides, including the largest f-element polyarsenide known to date.

## Graphical abstract

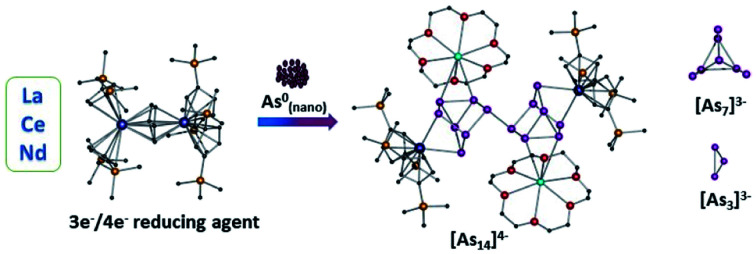

## Introduction

Zintl anion and Zintl cluster chemistry is a traditional topic in inorganic chemistry.^1^ However, recent spectacular new findings did not only result in new structural motifs but also shed some light into the formation of nanomaterials.^2–4^ For group 15 Zintl ions, the research on molecular coordination compounds is heavily focused on phosphorus and transition metals or main group elements.^5–8^ This can be ascribed to the availability of the molecular and soluble phosphorus allotrope P_4_ (white phosphorus), which allows the synthesis of often unpredictable and diverse phosphorus-containing compounds.^9^ Examples for the activation of P_4_ are the formation of P_4_^2−^ and other rings such as P_5_^−^ and P_6_.^5–7,10^ Also the opening of one edge of the P_4_ tetrahedron to form butterfly-type structures is known.^11–15^ While most of these reactions were reported with main group and transition metals, f-elements were less investigated and focused more on the lighter homologue nitrogen.^16–19^ In contrast, the heavier molecular congener As_4_ is inconvenient to synthesise, highly prone to decompose into its thermodynamically stable modification (gray arsenic) and extremely photosensitive.^20^ Therefore, only freshly prepared As_4_ solutions can be used for synthetic purposes.^21^ Unfortunately, even the latter suffer from rapid decomposition to gray arsenic and – as a consequence – a non-quantifiable As_4_ concentration. This is a major drawback for the synthesis and reproduction of unexpected polyarsenides. To circumvent these drawbacks, materials that can capture, stabilize, and release intact As_4_ tetrahedra were extensively investigated and designed.^22–24^ Scheer and co-workers developed a storage system for white phosphorus and yellow arsenic. This system uses porous activated carbon, in which intact As_4_ molecules were reversibly captured and released for the synthesis of transition metal complexes.^25^ With the synthesis of arsenic nanoparticles by reduction of AsI_3_ with lithium naphthalenide, we presented a different approach for this problem only recently, which allows the stoichiometric usage of an elemental As source.^26^ Starting from the nanoscale solid-state material (As^0^_nano_, *d* = 7.2 ± 1.8 nm) a variety of new f-element arsenic compounds as well as aluminium arsenic clusters have been successfully synthesised so far.^26–28^

In general, molecular arsenic Zintl ions of the lanthanides were only reported for Sm. In all these reactions the SET pathway from Sm(ii) to Sm(iii) was applied for the synthesis.^29^ Considering potential applications (lanthanide pnictide compounds have been discussed as potential thermoelectrical devices, transparent electrical contacts or solar cells)^30–32^ and in terms of possible optical and magnetic properties, this restriction to samarium compounds within 4f-element chemistry is a strong limitation.

**Fig. 1:**
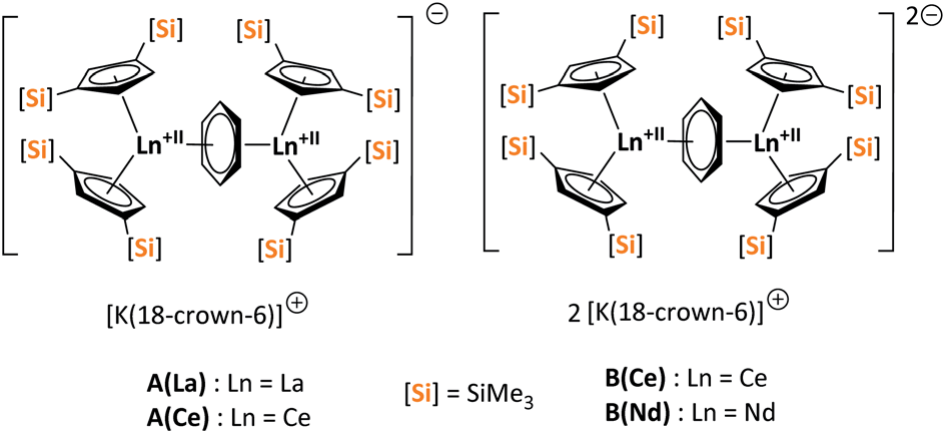
Three- (A(La), A(Ce)) and four- (B(Ce), B(Nd)) electron reducing agents, featuring non-classical divalent lanthanides ([Si] = SiMe_3_).^33,34^

Herein, we report a new pathway towards organo-f-element arsenic Zintl ions beyond Sm by combining the solid-state material As^0^_nano_ with the high redox potentials of various molecular non-classical divalent lanthanide compounds. For this purpose, the non-classical divalent lanthanide three electron reducing agents ([K(18-crown-6)][(
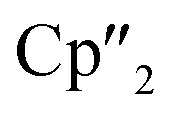
Ln)_2_(μ-η^6^:η^6^-C_6_H_6_)], Ln = La, Ce) (A) and four electron reducing agents ([K(18-crown-6)]_2_[(
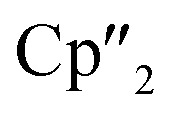
Ln)_2_(μ-η^6^:η^6^-C_6_H_6_)], Ln = Ce, Nd) (B) were employed (Fig. 1).^33–35^

## Results and discussion

**Scheme 1:**
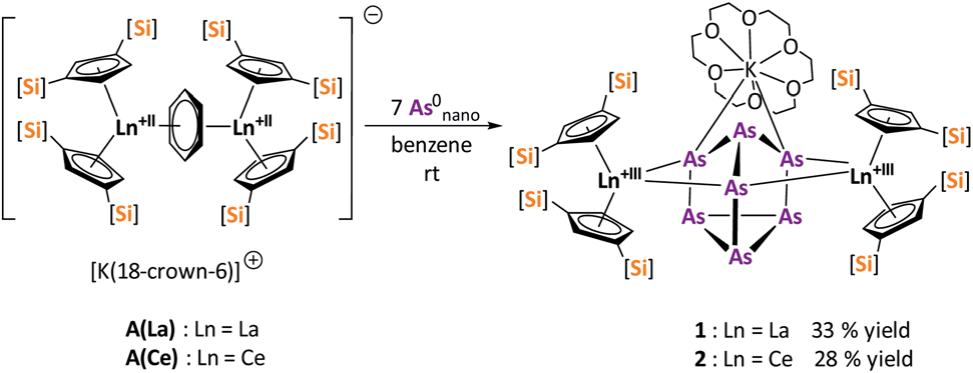
Synthesis of compounds 1 and 2.

**Fig. 2:**
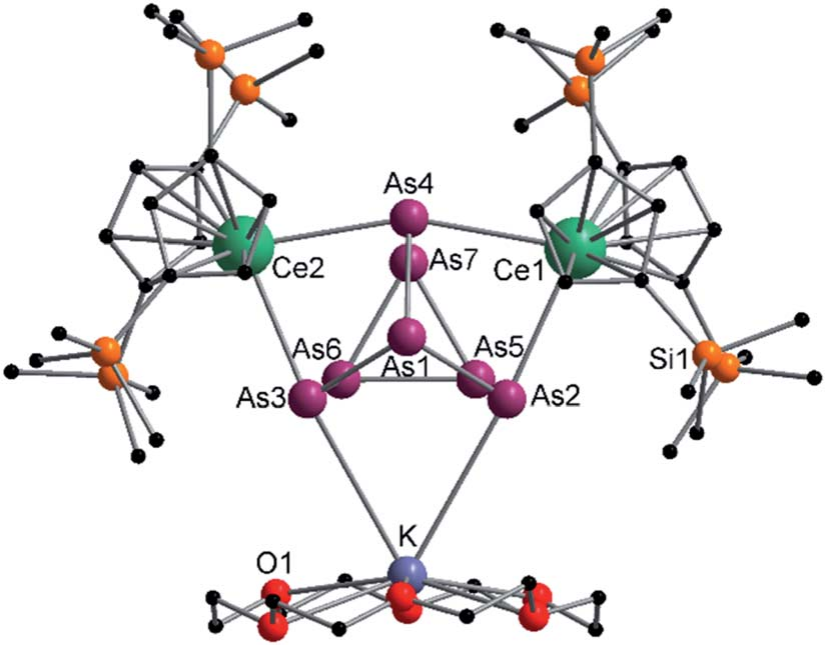
Molecular structure of 2 in the solid state. Solvent molecules, and hydrogen atoms are omitted for clarity. For bond lengths and angles see ESI (Fig. S9‡).

Reduction of As^0^_nano_ with the three electron reducing agents A(La) and A(Ce) resulted in the organo-lanthanide-polyarsenides [{K(18-crown-6)}(
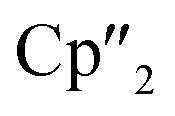
Ln)_2_(μ_3_-η^2^:η^2^:η^2^-As_7_)] (Scheme 1, Ln = La (1), Ce (2)) in yields of 33% for 1 and 28% for 2. Within these compounds an As_7_^3−^ Zintl anion with a nortricyclene structure forms the core of compounds 1 (Fig. S6‡) and 2 (Fig. 2). For charge balance, [K(18-crown-6)]^+^ and two 
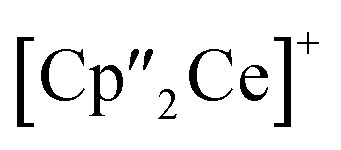
 fragments are coordinated by the equatorial arsenic atoms As2, As3 and As4 of the polyanion, each of which is bonded to two neighboring As atoms and thus negatively charged according to the 8-N rule. While a norbornadiene like As_7_^3−^ structure is known for Sm compounds, 1 and 2 featuring a nortricyclic structural motif in molecular f-element chemistry for the first time.^36^ A comparison of the 3-electron reducing agents A(Ce) and A(La) with the 1-electron reducing agent 
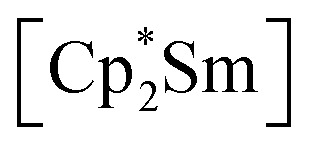
 at room temperature shows fundamental differences. While treatment of As^0^_nano_ with 
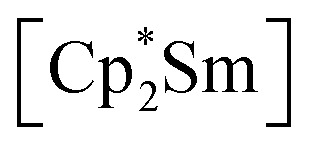
 leads to an As_2_^2−^ moiety compounds 1 and 2 feature the As_7_^3−^ scaffold.^26^ Furthermore, while the product formation in the presence of 
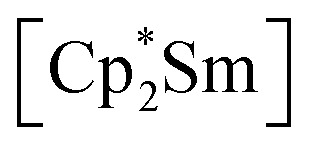
 is temperature dependent,^26^ the formation of 1 and 2 occurs also at elevated temperature.

It should be kept in mind that the solid-state material As^0^_nano_ is gray arsenic, which features a polymeric structure. Thus, the selective formation of the As_7_^3−^ cages is a complicated process, which requires several steps of bond breaking and bond formation.

Since compound 1 and 2 exhibit the same structural motif, only the molecular solid-state structure of compound 2 is discussed in detail here. The As–As bond lengths within the As_7_^3−^ unit are longest on average for the uncharged basal arsenic atoms As5, As6 and As7 (As5–As6 2.4654(5), As5–As7 2.4717(5), As6–As7 2.4805(5) Å). Compared to these, the bonds towards the charged arsenic atoms tend to be shortened, with those towards the apical and uncharged As1 being in average slightly longer than those towards the As5–As6–As7 plane (As1–As2 2.4101(5), As1–As3 2.4129(5), As1–As4 2.4777(5) Å *vs.* As2–As5 2.3877(5), As3–As6 2.3845(5), As4–As7 2.4010(5) Å). In contrast to symmetrically coordinated As_7_^3−^ units, *e.g.*, [(Li{dme})_3_As_7_] or K_3_As_7_,^37,38^ there are slight deviations in the bonding parameters from a symmetrical setup in the As_7_^3−^ core of compounds 1 and 2 due to the asymmetrical coordination with the various cationic fragments. The observed Ce–As bond lengths (Ce1–As2 3.0460(4), Ce1–As4 3.1053(4), Ce2–As3 3.0412(4), Ce2–As4 3.0897(4) Å) match with previous observations.^33^ The K–As distances are relatively long at ∼3.76 Å, which suggests weak coordination by the [K(18-crown-6)]^+^ fragment. Nevertheless, they are in a range observed for other potassium polyarsenides (*e.g.* 3.19–3.84 Å in [K(2.2.2-cryptand)]_2_(KAs_7_)).^39,40^

**Scheme 2:**
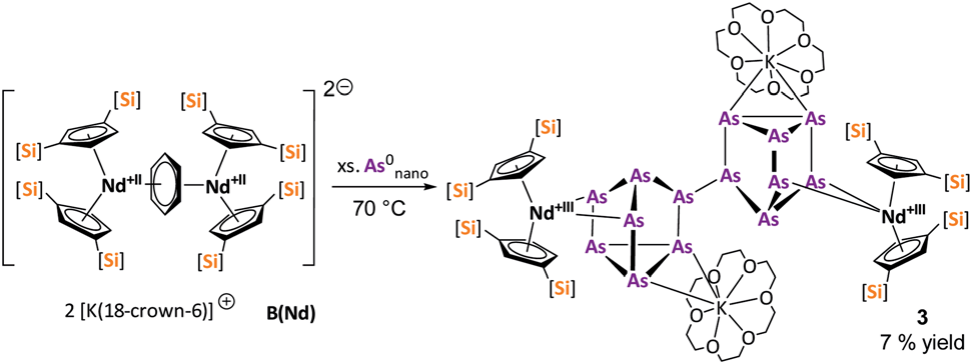
Clean isolation of compound 3.

After the successful application of the 3-electron reducing agents A(La) and A(Ce) in the activation of nanoscale arsenic, we felt challenged to treat As^0^_nano_ with 4-electron reducing agents in the reduction process. For comparison, we reacted the closely related compounds B(Ce) and B(Nd) with As^0^_nano_. This allows to maintain the same steric influence of the reducing agents to have an unobstructed view of the influence of the different reduction processes (three *vs.* four electrons). The reaction of both with As^0^_nano_ at room temperature resulted in a mixture of different products in the subsequent crystallization. However, by changing the reaction conditions upon usage of B(Nd) (excess of As^0^_nano_ and prolonged heating) it was possible to isolate [{K(18-crown-6)}_2_(
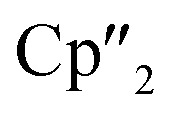
Nd)_2_(*μ*_4_-η^2^:η^2^:η^2^:η^2^-As_14_)] (3) exclusively but in low yields of 7% (Scheme 2).

**Fig. 3:**
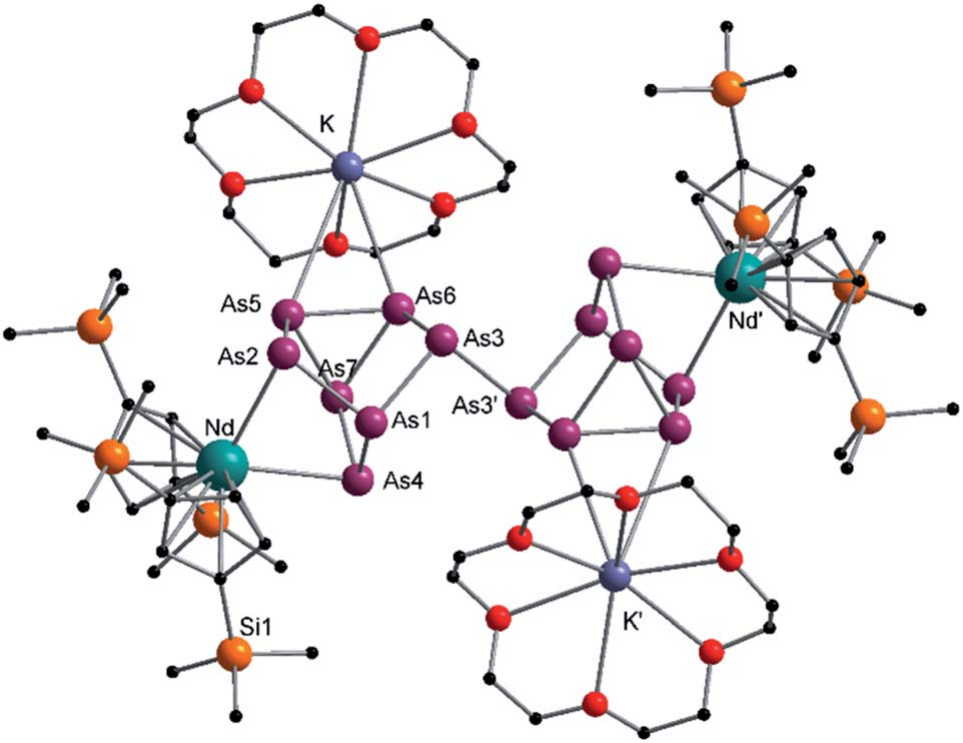
Molecular structure of 3 in the solid state. Solvent molecules and hydrogen atoms are omitted for clarity. Only one part of the positional disordered [K(18-crown-6)]^+^ is depicted. For both parts, bond lengths and angles see ESI (Fig. S10‡).

Compound 3 represents the largest organo-lanthanide-polyarsenide known to date. The central As_14_^4−^ unit formally consists of two covalently linked As_7_^2−^ units (Fig. 3).

Considering that As_7_^2−^ radicals are a known species,^41^ the formation of 3 can be explained *via* the radical recombination of two As_7_^2−^ units. The newly formed As–As bond, linking the two As_7_^2−^ units, of 2.4522(14) Å is in the range of a single bond (*e.g. ca.* 2.44 Å in carbene-stabilized diarene)^42^ and comparable to the bonds between the basal arsenic atoms As5, As6, and As7 (2.4516(10)–2.4580(11) Å). Analogous to the previously obtained As_7_^3−^ units, the As–As bonds involving the charged equatorial arsenic atoms As2 and As4 are the shortest. However, in contrast to the nortricyclene structures of 1 and 2, the [K(18-crown-6)]^+^ fragments are not coordinated by the equatorial and charged As atoms but by the basal ones, presumably for steric reasons. Additionally, they are disordered over two positions each (see Fig. S8‡). The 
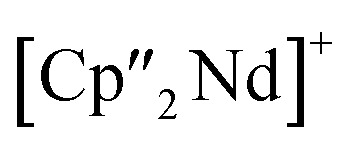
 cations are coordinated by the two respective charged equatorial arsenic atoms (Nd–As2 3.0506(7) Å, Nd–As4 3.0673(7) Å), with bond lengths comparable to the literature.^33^ In general, As_14_^4−^ Zintl anions are uncommon. There is one iron species, [K(dme)_2_]_2_[(Cp*Fe)_2_(μ,η^2:2:2:2^-As_14_)], which formally consists of two As_7_-norbonadiene motifs connected by means of an additional As–As bond.^43^ This is in contrast to 3, where two covalently linked As_7_-nortricyclene units form the As_14_^4−^ scaffold. The central motif of 3 is just reported from the solid state compound [Rb(18-crown-6)]_4_As_14_·6 NH_3_ and not known in molecular f-element chemistry.^44^

As an interim conclusion, we note that the formation and isolation of the As_7_^3−^ and As_14_^4−^ species by activation of nanoscale gray arsenic depends on the reducing agent used (3 *vs.* 4 electron reducing agents) and the reaction conditions. Moreover, the formation of the As_7_^3−^ and As_14_^4−^ species requires several steps of bond breaking and bond formation of gray arsenic. Thus, the formation of the well-characterized species 1–3 in one step seems to be unlikely. This can be seen by either using B(Ce) as a reducing reagent or by carrying out the reaction with B(Nd) at room temperature (note: 3 was formed at elevated temperature). In both cases mixtures of products were obtained. These could not be fully characterized as their separation failed due to similar solubility and identical appearance. However, their formation allows further insights into the complicated reduction process of As^0^_nano_ as a solid-state material. Therefore, the hereby obtained compounds should be briefly discussed in the following section.

**Scheme 3:**
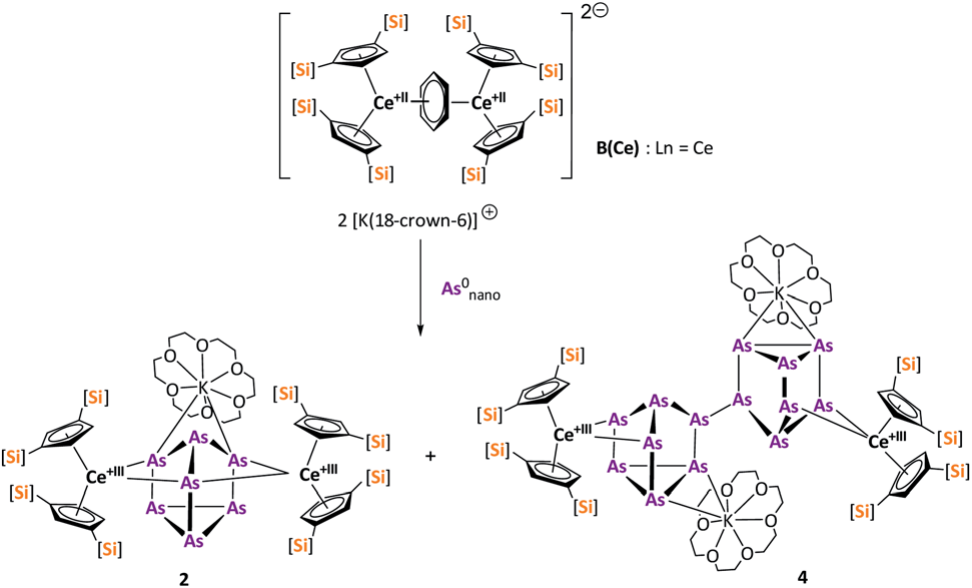
Synthesis of compounds 2 and 4.

**Fig. 4:**
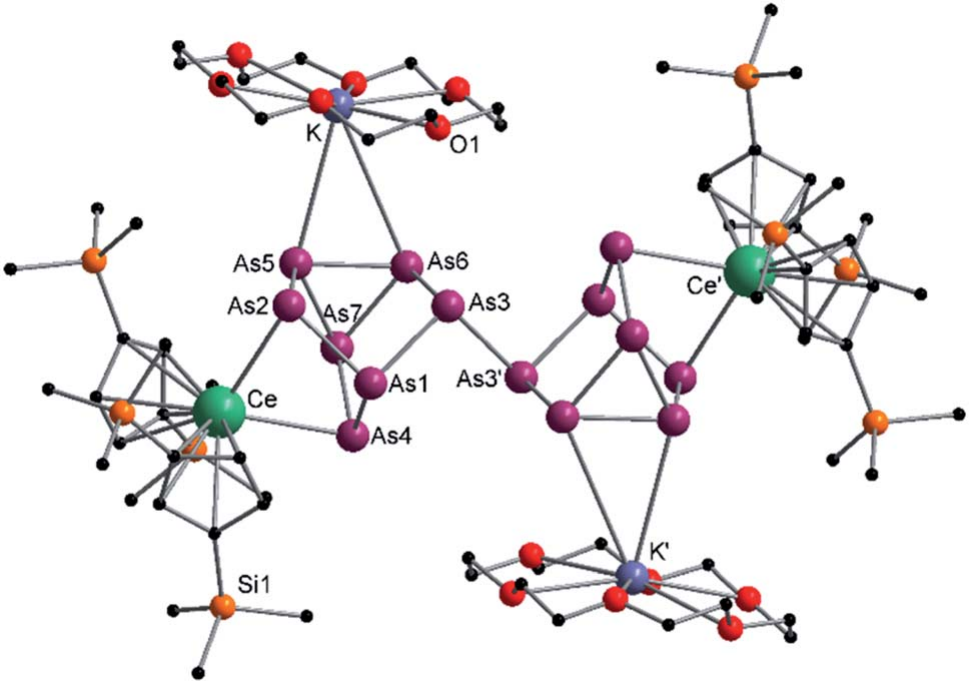
Molecular structure of 4 in the solid state. Solvent molecules and hydrogen atoms are omitted for clarity. Only one part of the positional disordered [K(18-crown-6)]^+^ is depicted. For both parts, bond lengths and angles see ESI (Fig. S11‡).

The activation of As^0^_nano_ with B(Ce) at room temperature resulted in two different products (Scheme 3). In addition to crystals of compound 2, which was already obtained when A(Ce) was used, crystals of the As_14_^4−^ species [{K(18-crown-6)}_2_(
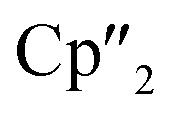
Ce)_2_(μ_4_-η^2^:η^2^:η^2^-As_14_)] (4) (Fig. 4) were isolated, representing the Ce analogue of 3. As observed for 3, the newly formed As–As bond of 2.4468(13) Å is in the range of the bonds of the also uncharged basal arsenic atoms As5, As6, and As7 (2.4490(10)–2.4661(9) Å).

All other bond lengths are in accordance with 3 as well. The 
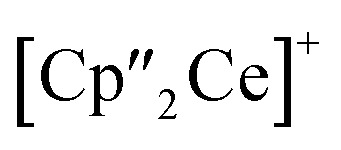
 cations also coordinate to the two respective charged equatorial arsenic atoms (Ce–As2 3.0598(8) Å, Ce–As4 3.0417(7) Å), with bond lengths comparable to the corresponding bonds in 2. Analogous to 3, the [K(18-crown-6)]^+^ fragments are disordered over two positions (Fig. S11‡).

**Scheme 4:**
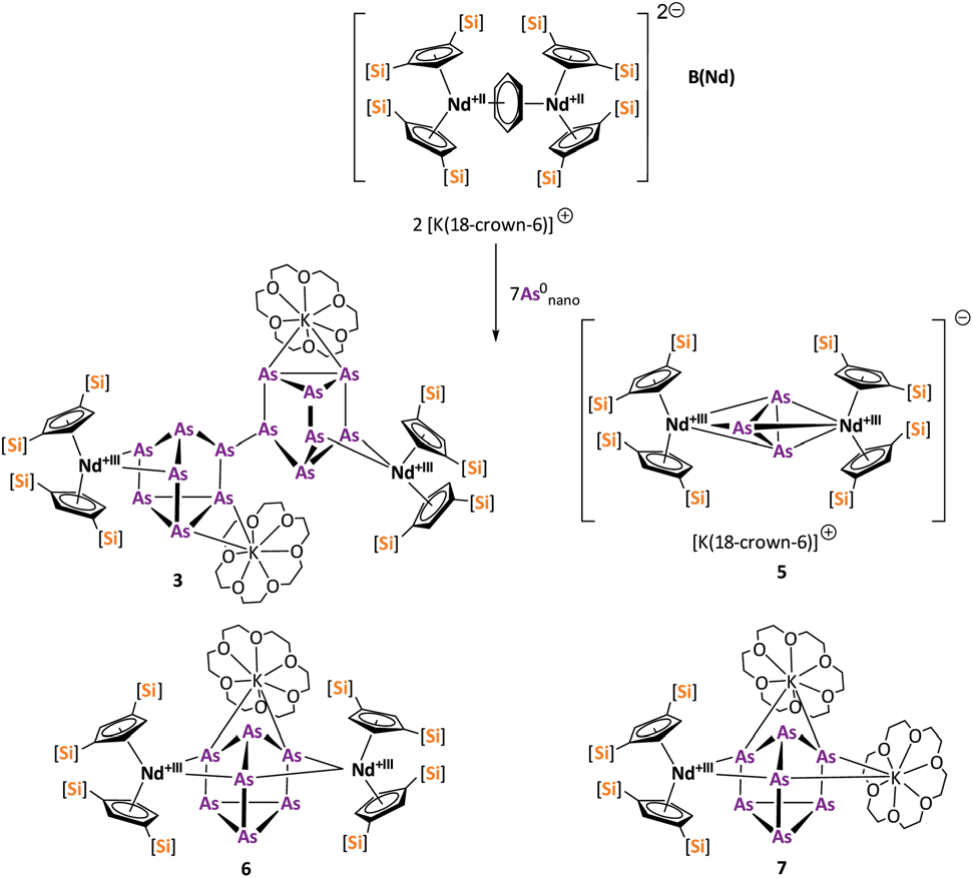
Synthesis of compounds 3, 5, 6 and 7.

In contrast to B(Ce), the use of the 4-electron Nd reducing agent B(Nd) at room temperature resulted in mixture of even more products upon crystallization (Scheme 4). As result, four different species were formed under these conditions, indicating an influence of the different used lanthanides and their reactivity on the reaction. The obtained products are the As_3_^3−^ species [K(18-crown-6)][(
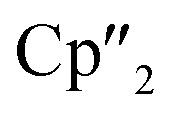
Nd)_2_(μ-η^3^:η^3^-As_3_)] (5), two compounds with an As_7_^3−^ motif [{K(18-crown-6)}(
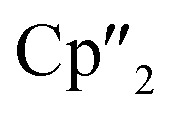
Nd)_2_(μ_3_-η^2^:η^2^:η^2^-As_7_)] (6) and [{K(18-crown-6)}_2_(
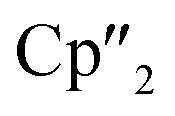
Nd)(μ_3_-η^2^:η^2^:η^2^-As_7_)] (7) as well as the isolable As_14_^4−^-compound (3).

**Fig. 5:**
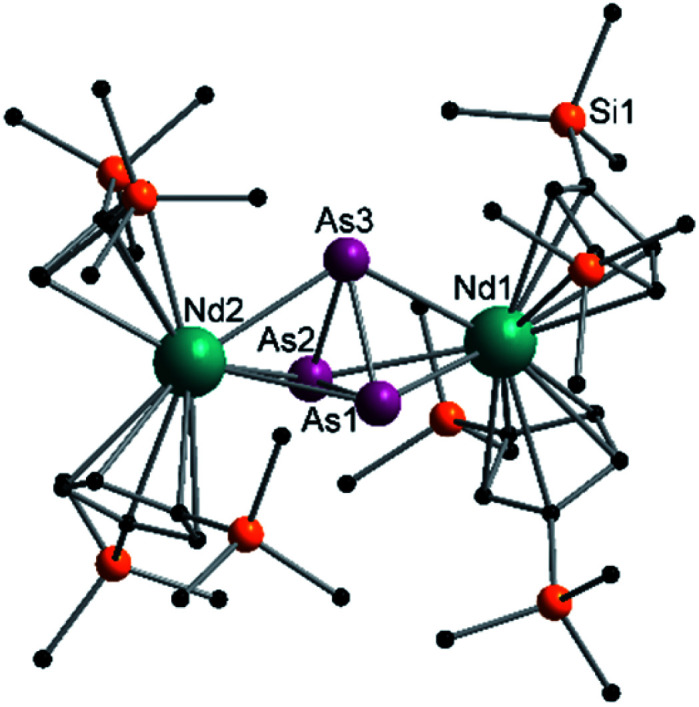
Molecular structure of 5 in the solid state. Solvent molecules, counter cation [K(18-crown-6)]^+^ and hydrogen atoms are omitted for clarity. For bond lengths and angles see ESI (Fig. S12‡).

Within the mixture obtained at room temperature, [K(18-crown-6)][(
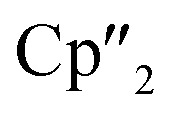
Nd)_2_(μ-η^3^:η^3^-As_3_)] (5) (Fig. 5) features the smallest of the organo-lanthanide-polyarsenides obtained here (As_3_^3−^ unit) and – similar to [(
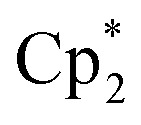
Sm)_2_(μ-η^2^:η^2^-As_2_)] from the activation of As^0^_nano_ by SET – may be regarded as an intermediate in the formation of the larger polyarsenides.^26^ The 
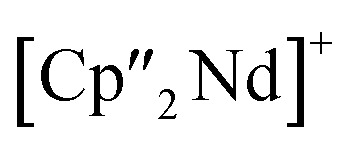
 fragments are slightly offset from each other and both η^3^-coordinated by the central As_3_^3−^ moiety, which in turn forms an equilateral triangle. The Nd–As bond lengths range from 2.9311(7) to 3.0481(7) Å and are thus comparable to the shortest Nd–As distance in [K(18-crown-6)][
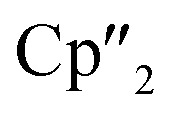
Nd(*μ-η*^4^:*η*^4^-As_5_)FeCp*] (2.9258(4) Å).^33^ The As–As distances (2.4198(7)–2.4388(8) Å), all of approximately equal length, are in the range of single bonds.^42^ Additionally, they are comparable to As_3_^3−^ units in solid state compounds (2.43–2.47 Å in CsAs).^38^ While an example of an uranium complex with such an As_3_^3−^ unit is reported for the actinides,^45^ this has not been accessible for the lanthanides so far.

**Fig. 6:**
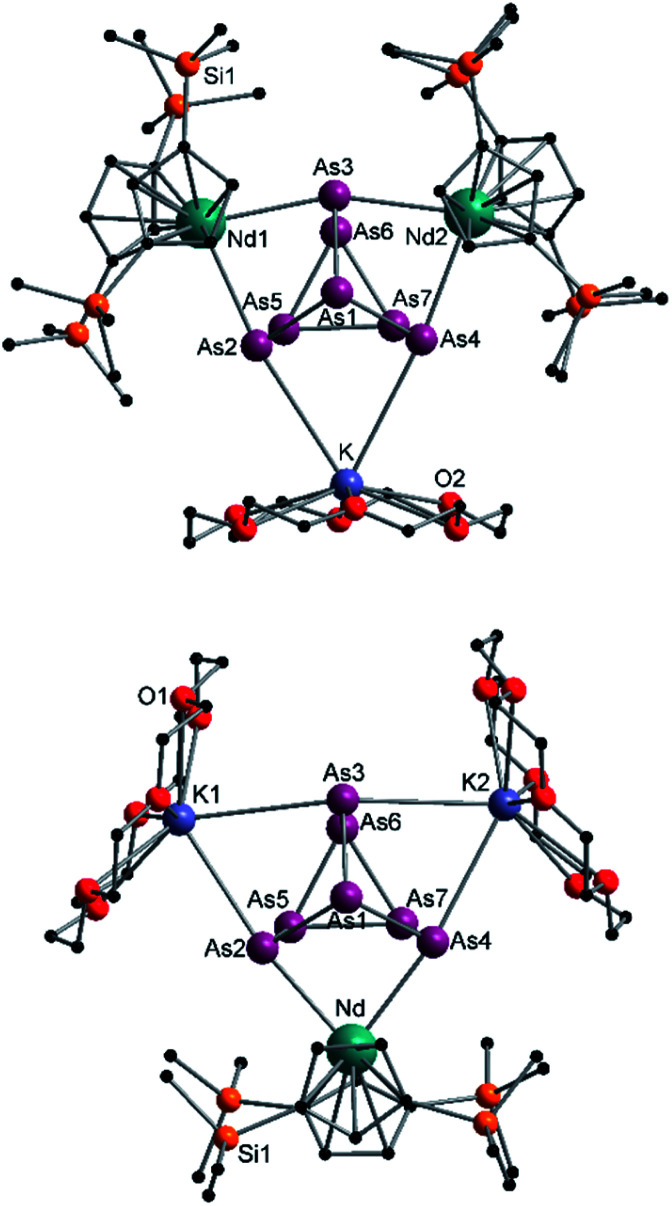
Molecular structures of 6 (top) and 7 (bottom) in the solid state. Solvent molecules and hydrogen atoms are omitted for clarity. For bond lengths and angles see ESI (Fig. S13 and S14‡).

In addition, two different compounds with an As_7_^3−^ structural motif were also found in the mixture (Fig. 6). Here, [{K(18-crown-6)}(
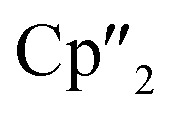
Nd)_2_(μ_3_-η^2^:η^2^:η^2^-As_7_)] (6) represents the Nd analogue to 2. The observed bond lengths are all comparable to the latter. In contrast, [{K(18-crown-6)}_2_(
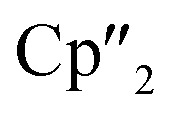
Nd)(μ_3_-η^2^:η^2^:η^2^-As_7_)] (7) shows an altered composition, since the charge balance takes place by means of a 
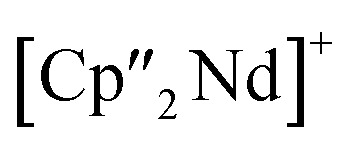
 fragment and two [K(18-crown-6)]^+^ units (*vice versa* for 1, 2, and 6). All bond lengths are within the previously observed ranges.

Finally, the last compound that can be obtained in the mixture is the already described As_14_^4−^ compound 3. As mentioned above, this compound could also be obtained in pure form in the presence of an excess of As^0^_nano_ and by prolonged heating (Scheme 2).

## Conclusions

In summary, we have demonstrated that the solid state material As^0^_nano_ can be activated *via* 3- and 4-electron reducing agents of the early non-classical divalent lanthanides to obtain a variety of new molecular organo-lanthanide-polyarsenides. This significantly extends the bridge from solid-state arsenic to molecular f-element polyarsenides, contributing to a better understanding of the formation and properties of such polyarsenide materials.

On the one hand, the clean formation of an As_7_^3−^ Zintl anion with a nortricyclic structure in 1 and 2 is observed by using the 3-electron reducing agents A(La) and A(Ce). Although lanthanide compounds with As_7_^3−^ Zintl anions have already been reported, the nortricyclane structural motif was previously unknown in this chemistry. On the other hand, the formation the As_14_^4−^ Zintl species 3 as sole isolable product is seen by applying the 4-electon reducing agents B(Nd) at elevated temperature. Compound 3 represents the largest known organo-lanthanide-polyarsenides to date. In between these boundaries, mixtures of various compounds with a polyarsenide as central motif were obtained. These results show that the formation of sophisticated structures directly out of nanoscale gray arsenic, which is a kind of polymer, is a complex process with various intermediates. Only careful tuning of the reaction conditions and the use of an optimized reducing reagent leads to isolable and unprecedented products.

## Data availability

All synthetic protocols, spectroscopic data, supplementary figures and tables, and detailed crystallographic information can be found in the ESI.‡ Crystallographic data are available *via* the Cambridge Crystallographic Data Centre (CCDC): 2094692–2094698.

## Author contributions

All authors have given approval to the final version of the manuscript. N. R. and A. H. synthesized and analyzed all compounds. L. M conducted X-ray experiments. PWR originated the idea, supervised the work, and interpreted the results. All authors contributed to the preparation of the manuscript.

## Conflicts of interest

There are no conflicts to declare.

## Supplementary Material

SC-013-D1SC05797A-s001

SC-013-D1SC05797A-s002
