# Supplementary material for: From a nanoparticular solid-state material to molecular organo-f-element-polyarsenides
Source: Chem Sci. 2022 Feb 4;13(12):3363–8. doi: 10.1039/d1sc05797a (PMC8943856; doi:10.1039/d1sc05797a)
Supplement: SC-013-D1SC05797A-s001 [file SC-013-D1SC05797A-s001.pdf]

Electronic Supplementary Material (ESI) for Chemical Science.
This journal is © The Royal Society of Chemistry 2022

Supporting Information

From a Nanoparticular Solid-State Material to Molecular Organo-f-Element-Polyarsenides

Niklas Reinfandt†, Adrian Hauser†, Luca Münzfeld, Peter W. Roesky*

Institute of Inorganic Chemistry
Karlsruhe Institute of Technology (KIT)
Engesserstr. 15, Geb. 30.45, D-76131 Karlsruhe, Germany.
E-mail: roesky@kit.edu
† These authors contributed equally to this work.

General Methods:

All manipulations of water- and air-sensitive compounds were performed with exclusion of moisture and oxygen in flame-dried Schlenk-type glassware either on a dual manifold Schlenk line, interfaced to a high vacuum (10^{-3} mbar) line or in an argon-filled MBraun glove box. Tetrahydrofuran was distilled under nitrogen from potassium benzophenoneketyl before storage in vacuo over $LiAlH_4$. Hydrocarbon solvents were dried by using an MBraun solvent purification system (SPS 800), degassed and stored *in vacuo* over $LiAlH_4$. Elemental analysis were carried out with an Elementar Vario Micro cube. IR spectra were obtained on a Bruker Tensor 37 spectrometer equipped with a room temperature DLaTGS detector and a diamond ATR (attenuated total reflection) unit.

As^0_{nano},[1] [K(18-crown-6)][(Cp''$_2$La)$_2$(μ-η^6:η^6-C_6H_6)] (**A(La)**),[2, 3] [K(18-crown-6)][(Cp''$_2$Ce)$_2$(μ-η^6:η^6-C_6H_6)] (**A(Ce)**), [K(18-crown-6)]$_2$[(Cp''$_2$Ce)$_2$(μ-η^6:η^6-C_6H_6)] (**B(Ce)**) and [K(18-crown-6)]$_2$[(Cp''$_2$Nd)$_2$(μ-η^6:η^6-C_6H_6)]] (**B(Nd)**) were prepared following literature procedures.[4]

Synthesis of [{K(18-crown-6)}(Cp''$_2$La)$_2$(μ_3-η^2:η^2:η^2-As$_7$)] (1):

Toluene (10 mL) was condensed onto a mixture of **A(La)** (100.0 mg, 0.067 mmol, 1.00 equiv.) and As$^0_{nano}$ (35.0 mg, 0.47 mmol, 7.00 equiv.). The resulting reaction mixture suspended for 4 h in an ultrasonic bath and afterwards stirred for 96 h at RT. All volatiles were removed under reduced pressure and the remaining solid was extracted with 10 mL benzene. Crystals of **1** were obtained by slow evaporation of the solvent at ambient temperature and carefully washed with cold benzene.

Yield: 42.0 mg (0.022 mmol, 33 %).

NMR: ^{1}H NMR (400.30 MHz, THF-*d$_8$*, 300 K) δ/ppm = 6.68-6.45 (m, 12 H, C*H$_{Cp}$*), 3.65 (s, 24 H, C*H$_2$*), 0.32 (s, 72 H, C*H$_3$*). ^{13}C{^{1}H} NMR (100.67 MHz, THF-*d$_8$*) δ/ppm = 137.0 (s, *Cp*), 131.4 (s, *Cp*), 119.8 (s, *Cp*), 71.4 (s, *18-crown-6*), 2.0 (s, *TMS*), 1.9 (s, *TMS*).

IR (ATR): $\tilde{\nu}$ [cm^{-1}] = 2948 (s), 2890 (s), 1434 (w), 1351 (w), 1316 (vw), 1243 (s), 1107 (v), 1076 (s), 962 (m), 919 (m), 823 (vs), 780 (m), 749 (s), 688 (w), 637 (m), 475 (w).

Anal. calcd. (%) for [$C_{56}H_{108}As_7La_2KO_6Si_8$] (1943.51 g/mol): C: 34.61, H: 5.60; **found** (%): C: 34.83, H: 4.89.

Synthesis of [{K(18-crown-6)}(Cp''$_2$Ce)$_2$(μ_3-η^2:η^2:η^2-As$_7$)] (2):

Toluene (10 mL) was condensed onto a mixture of **A(Ce)** (100.0 mg, 0.067 mmol, 1.00 equiv.) and As$^0_{nano}$ (35.0 mg, 0.47 mmol, 7.00 equiv.). The resulting reaction mixture suspended for 4 h in an ultrasonic bath and afterwards stirred for 96 h at RT. All volatiles were removed under reduced pressure and the remaining solid was extracted with 10 mL benzene. Crystals of **2** were obtained by slow evaporation of the solvent at ambient temperature and carefully washed with cold benzene.

Yield: 38.0 mg (0.019 mmol, 28 %).

IR (ATR): $\tilde{\nu}$ [cm^{-1}] = 2950 (s), 2890 (s), 1536 (vw), 1452 (w), 1351 (m), 1246 (s), 1108 (vs), 1076 (m), 962 (m), 918 (m), 824 (vs), 783 (m), 748 (s), 687 (w), 636 (m), 474 (w).

Anal. calcd. (%) for [$C_{56}H_{108}As_7Ce_2KO_6Si_8$] (1945.94 g/mol): C: 34.57, H: 5.59; **found** (%): C: 34.93, H: 5.03.

Owing to the paramagnetic nature of Ce(III), no meaningful NMR spectra were obtained (For ^{1}H NMR see Figure S3). For the calculation of X_M via the Evans method see Figure S4.

Synthesis of [{K(18-crown-6)}$_2$(Cp''$_2$Nd)$_2$(μ_4-η^2:η^2:η^2:η^2-As$_{14}$)] (3):

Toluene (10 mL) was condensed onto a mixture of **B(Nd)** (100.0 mg, 0.055 mmol, 1.00 equiv.) and As^0_{nano} (29.0 mg, 0.39 mmol, 7.00 equiv.). The resulting reaction mixture suspended for 4 h in an ultrasonic bath and afterwards stirred for 96 h at 70 °C. All volatiles were removed under reduced pressure and the remaining solid was extracted with 10 mL benzene. Yellowish crystals of **3** were obtained by slow evaporation of the solvent at ambient temperature and washed with cold benzene.

Yield: 10 mg (0.0036 mmol, 7 %).

IR (ATR): $\tilde{\nu}$ [cm^{-1}] = 2948 (s), 2888 (s), 1652 (vw), 1536 (w), 1470 (w), 1351 (m), 1247 (s), 1105 (vs), 961 (m), 923 (vw), 828 (vs), 749 (s), 684 (m), 636 (m), 474 (vw).

Anal. calcd. (%) for [$C_{68}H_{66}As_{14}K_2Nd_2O_{12}Si_8$] (2782.06 g/mol): C: 29.36, H 4.78; **found** (%): C: 29.87, H: 4.75.

Owing to the paramagnetic nature of Nd(III) and the low stability of the compound **3** upon redissolving its single crystals, no meaningful NMR spectra could be obtained.

Synthesis of [{K(18-crown-6)}(Cp''$_2$Ce)$_2$(μ_3-η^2:η^2:η^2-As$_7$)] (2) and [{K(18-crown-6)}$_2$(Cp''$_2$Ce)$_2$(μ_4-η^2:η^2:η^2:η^2-As$_{14}$)] (4):

Toluene (10 mL) was condensed onto a mixture of **B(Ce)** (100.0 mg, 0.055 mmol, 1.00 equiv.) and As^0_{nano} (29.1 mg, 0.39 mmol, 7.00 equiv.). The resulting reaction mixture suspended for 4 h in an ultrasonic bath and afterwards stirred for 96 h at 60 °C. All volatiles were removed under reduced pressure and the remaining solid was extracted with 10 mL benzene. A mixture of orange-reddish crystals of **2** and **4** was obtained by slow evaporation of the solvent at ambient temperature. Due to the similar solubility of the compounds, a separation was not possible.

Synthesis of [K(18-crown-6)][(Cp''$_2$Nd)$_2$(μ-η^3:η^3-As$_3$)] (5), [{K(18-crown-6)}(Cp''$_2$Nd)$_2$(μ_3-η^2:η^2:η^2-As$_7$)] (6), [{K(18-crown-6)}$_2$(Cp''$_2$Nd)(μ_3-η^2:η^2:η^2-As$_7$)] (7) and [{K(18-crown-6)}$_2$(Cp''$_2$Nd)$_2$(μ_4-η^2:η^2:η^2:η^2-As$_{14}$)] (3):

Toluene (10 mL) was condensed onto a mixture of **B(Nd)** (100.0 mg, 0.055 mmol, 1.00 equiv.) and As^0_{nano} (29.0 mg, 0.39 mmol, 7.00 equiv.). The resulting reaction mixture suspended for 4 h in an ultrasonic bath and afterwards stirred for 96 h at RT. All volatiles were removed under reduced pressure and the remaining solid was extracted with 10 mL benzene. A mixture of orange crystals of **3, 5, 6,** and **7** was obtained by slow evaporation of the solvent at ambient temperature. Due to the similar solubility of the compounds, a separation was not possible.

NMR Spectra:

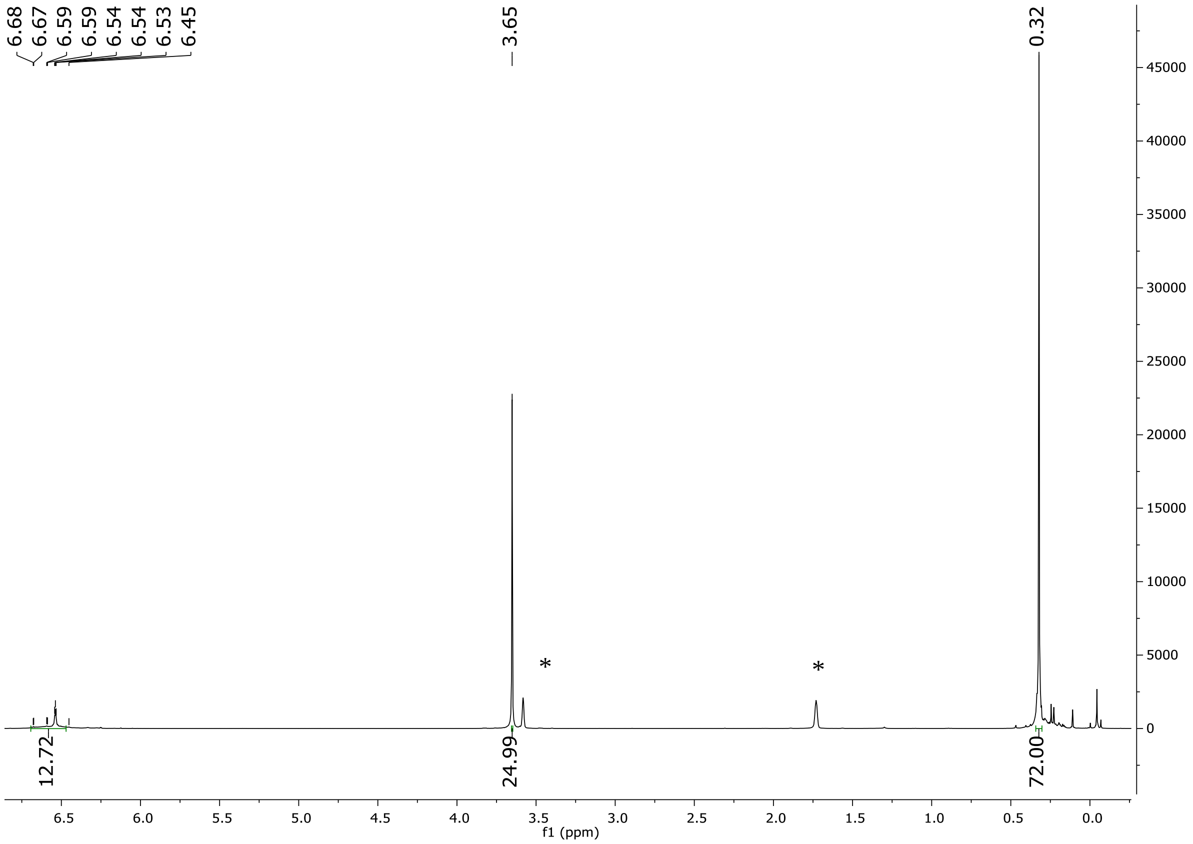

Figure S1: ^{1}H NMR of **1** in THF-*d8*. *, residual proton solvent signal.

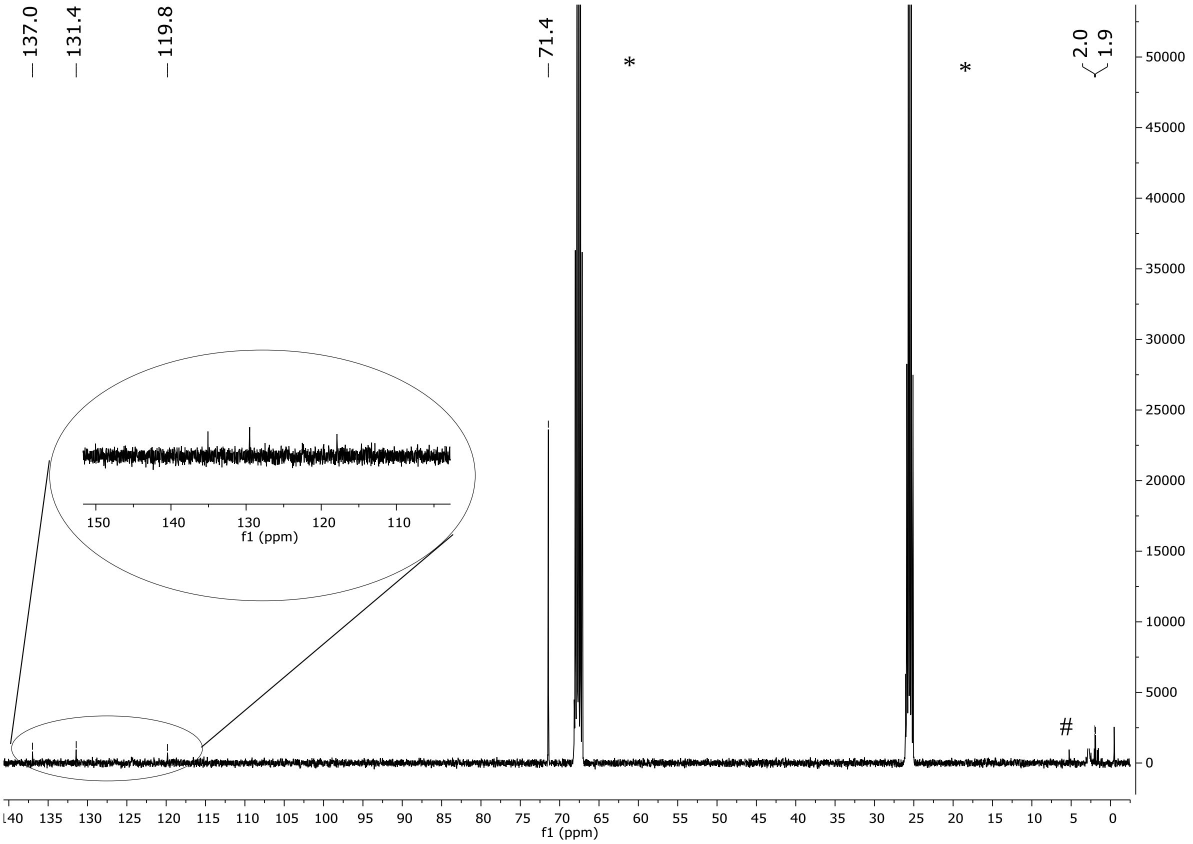

Figure S2: $^{13}C\{^{1}H\}$ NMR of **1** in THF-*d8*. *, solvent signal. #, unknown decomposition product.

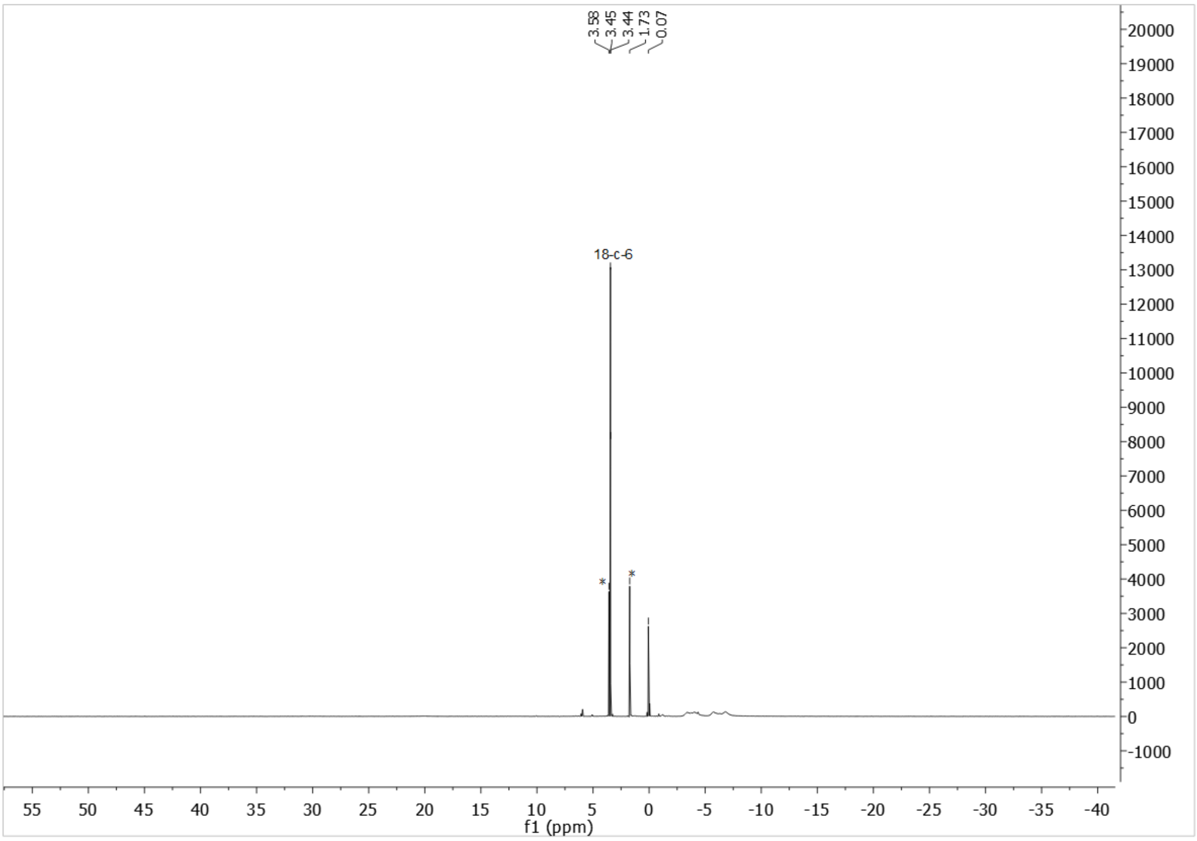

Figure S3: ^{1}H NMR of **2** in THF-d_8. *, residual proton solvent signal.

NMR Data applying the Evans Method (compound 2)

Evans Method:[5]

Unpaired electrons originating from a paramagnetic substance in solution will result in a change of the chemical shift of all the species in solution. As first presented by Evans *et al.* 1959, this allows the determination of the substance's magnetic susceptibility χ_M. NMR Data is obtained by preparing a NMR tube that contains a capillary of pure solvent (THF-d_8/THF = 50:1), with a solution of the paramagnetic substance **2** surrounding the capillary within the tube.

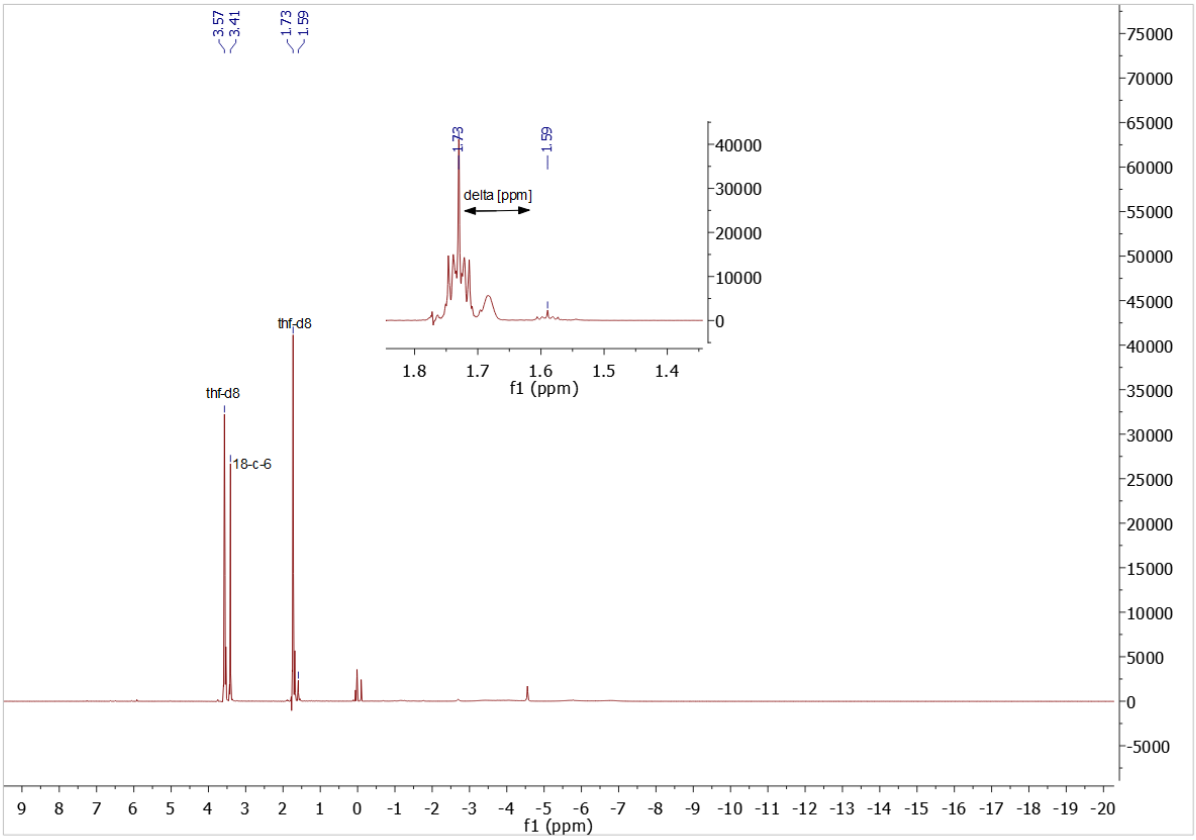

Figure S4: ^{1}H NMR spectrum of compound (**2**) in a mixture of THF-d_8/THF = 50:1 (400 MHz, 298 K). Additionally, the NMR tube contains a capillary containing the pure solvent mixture (THF-d_8/THF = 50:1). The substance's magnetic susceptibility χ_M can be determined by the difference between the shifted and the pure solvent resonance (Δ[ppm]). Due to the paramagnetic Ce(III) species only the 18-crown-6 signal can be detected and clearly assigned in the spectrum.

Calculation:

$$\Delta f\ (Hz) = \frac{\Delta[ppm] \cdot F}{10^6} = \frac{0.14 \cdot 400 \cdot 10^6\ Hz}{10^6} = 56\ \text{Hz}$$

$$\chi_M = \frac{3\Delta f}{4\pi F c} = \frac{3 \cdot 56\ Hz}{4\pi(400 \cdot 10^6\ Hz)\ (7.19 \cdot 10^{-6}\ mol \cdot mL^{-1})} = = 4.6 \cdot 10^{-3}\ \text{cm}^3\text{/mol}$$

Δf = frequency difference [Hz] between the shifted and the pure solvent resonance

F = spectrometer radiofrequency [Hz]

c = concentration of paramagnetic species [mol/mL]

(applied sample: m = 7 mg; M_W = 1945.94 g·mol^{-1}; V = 0.5 mL)

$$\mu = \sqrt{8(\chi_M T)} = \sqrt{8(4.6 \cdot 10^{-3} \cdot\ 298)} = \sqrt{11.08} = 3.32\ \mu_B$$

X_M = molar magnetic susceptibility [cm^3/mol]

T = temperature [K]

μ = magnetic moment, measured in units of Bohr magneton, μ_B = 9.27 · 10^{-24} J·T^{-1}

IR Spectra:

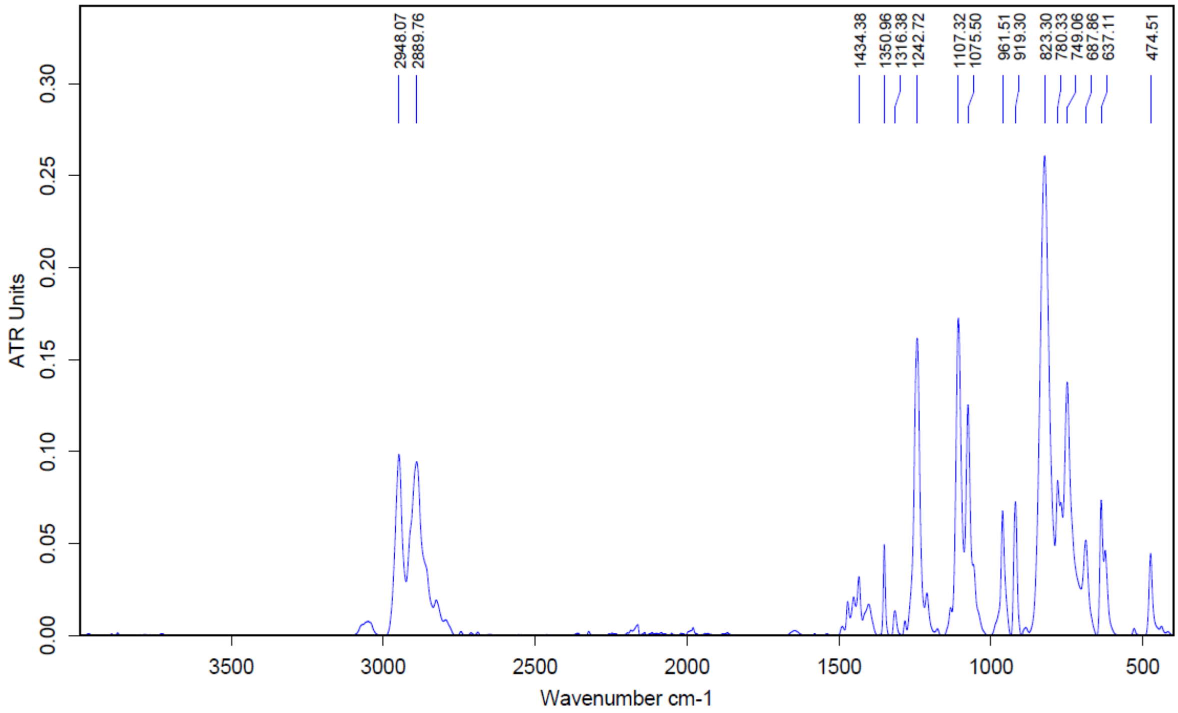

Figure S5: IR-Spectrum (ATR) of **1**.

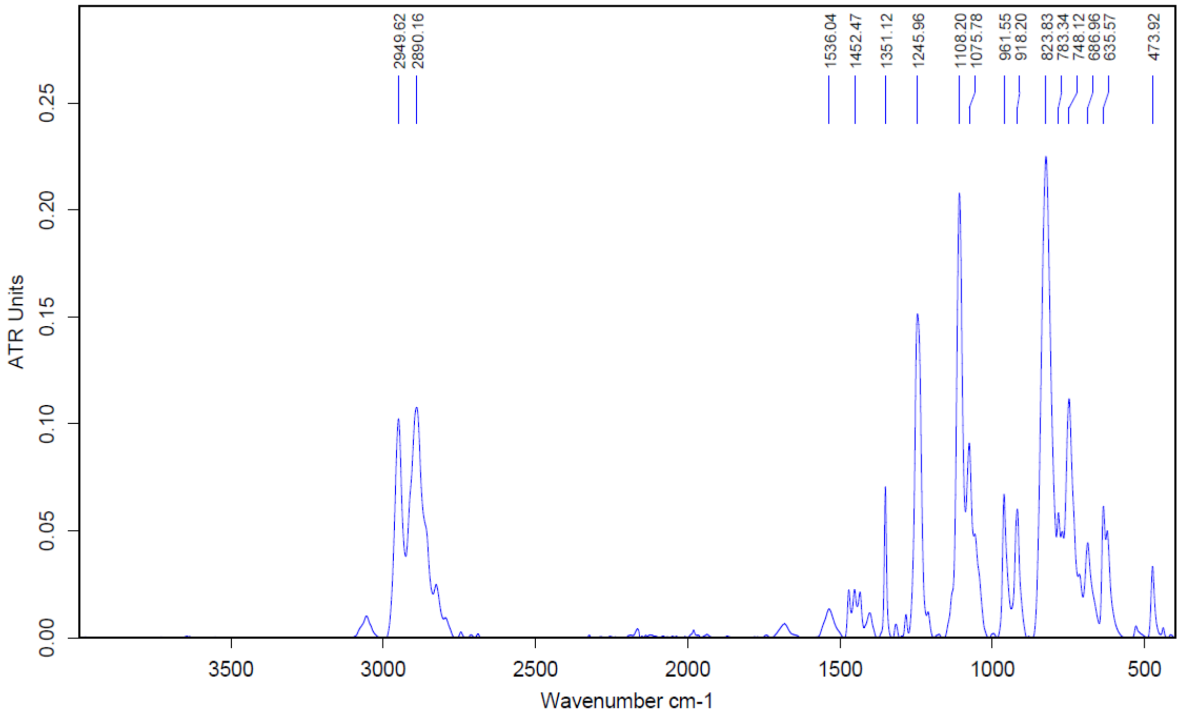

Figure S6: IR-Spectrum (ATR) of **2**.

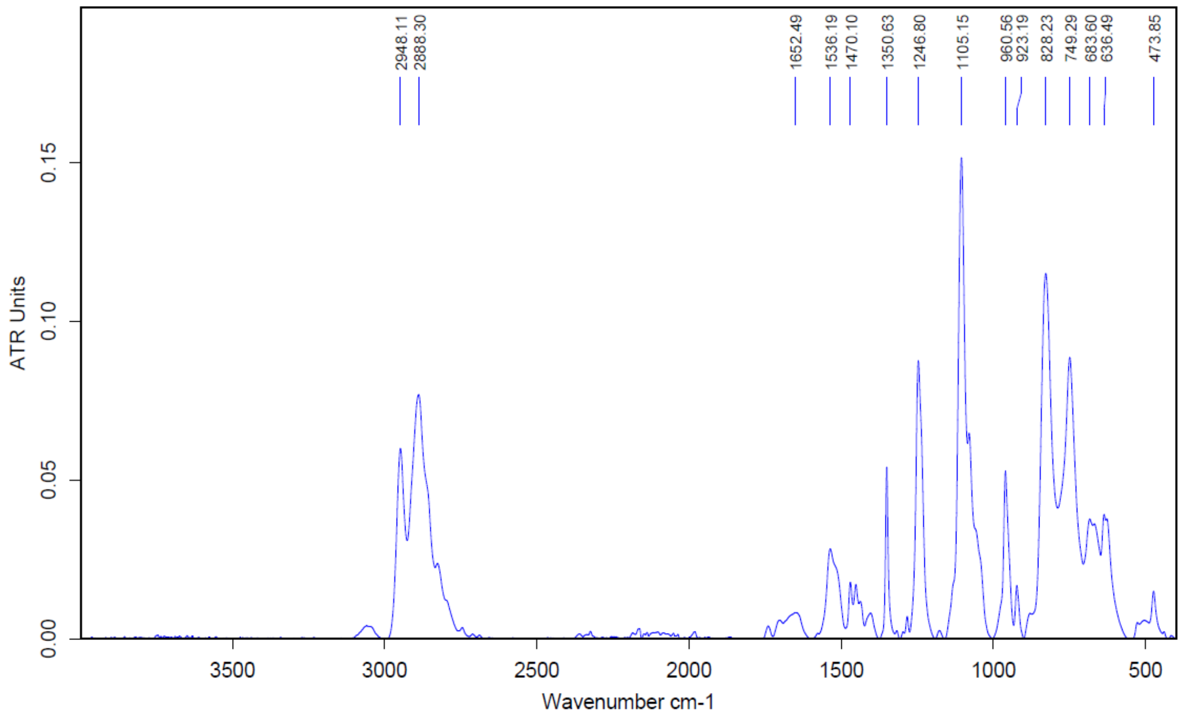

Figure S7: IR-Spectrum (ATR) of **3**.

X-ray Crystallographic Studies

Suitable crystals were selected under an optic microscope equipped with polarizing filters, covered in mineral oil (Aldrich) and mounted on a MiTeGen holder. The crystals were transferred directly to the cold stream of a STOE IPDS 2 (150 K) or STOE StadiVari (100 K) diffractometer, equipped with a Mo-sealed tube, MoGenix 3D HF or a Ga-MetalJet X-ray source.

All structures were solved using the programs SHELXS/T and Olex2 1.2.4,5,6.[6-9] The remaining non-hydrogen atoms were located from successive difference Fourier map calculations. The refinements were carried out by using full-matrix least-squares techniques on F2 by using the program SHELXL.

Specific comments on the structures discussed here are given below.

Crystallographic data (excluding structure factors) for the structures reported in this paper have been deposited with the Cambridge Crystallographic Data Centre as a supplementary publication no. 2094692-2094698. Copies of the data can be obtained free of charge on application to CCDC, 12 Union Road, Cambridge CB21EZ, UK (fax: (+(44)1223-336-033; email: deposit@ccdc.cam.ac.uk).

Note: All single crystals of compounds discussed here systematically showed differently pronounced signs of twinning. As the abundance of the minor domains was significantly lower compared to the main domain in all cases, this problem could not reasonably be accounted for by means of twin integration and application of suitable twin laws. For compounds **2** and **4** these problems could be mitigated by crystallization from toluene instead of benzene. However, the core structural motifs could be unambiguously solved and refined for all compounds obtained. Exact bond lengths and angles should be considered with care.

[{K(18-crown-6)}(Cp''$_2$La)$_2$(μ_3-η^2:η^2:η^2-As$_7$)] (1):

Crystals of compound **1** showed signs of twinning, leading to intense secondary and third set of q-peaks for all heavy atom positions. This causes all A- and B-level alerts in the checkCIF routine output. SIMU restraints were used to model disordered solvent molecules and one TMS group.

Alert level A

PLAT971_ALERT_2_A

Check Calcd Resid. Dens. 0.83A From La1 3.95 eA-3

Check Calcd Resid. Dens. 1.15A From La1 3.52 eA-3

Alert level B

PLAT971_ALERT_2_B

Check Calcd Resid. Dens. 1.32A From La2 3.50 eA-3

Check Calcd Resid. Dens. 1.12A From La2 2.65 eA-3

Check Calcd Resid. Dens. 1.25A From La1 2.51 eA-3

Author Response: The residual electron density is likely caused by untreated twinning. This leads to a second and third set of clearly visible q-Peaks for every heavy in the core structure. However, the chemical abundance of the twinned/disordered structure is too low, to be treated satisfactorily.

[{K(18-crown-6)}(Cp''$_2$Ce)$_2$(μ_3-η^2:η^2:η^2-As$_7$)] (2)

The solid-state structure of compound **2** features a disordered toluene molecule in the asymmetric unit. Moderate ISOR restraints were applied to model the disorder appropriately.

[{K(18-crown-6)}$_2$(Cp''$_2$Nd)$_2$(μ_4-η^2:η^2:η^2:η^2-As$_{14}$)] (3):

The solid-state structure of compound **3** features a heavily disordered [K(18-c-6)] fragment in the asymmetric unit. SIMU and SADI restraints were employed to appropriately model the disordered cationic moiety.

[{K(18-crown-6)}$_2$(Cp''$_2$Ce)$_2$(μ_4-η^2:η^2:η^2:η^2-As$_{14}$)] (4)

Crystals of **4** exhibit a positional disorder of the [K(18-c-6)] fragment and two toluene molecules. Both disorders were modelled using SIMU restraints.

[K(18-crown-6)][(Cp''$_2$Nd)$_2$(μ-η^3:η^3-As$_3$)] (5)

Compound **5** exhibits two disordered TMS groups and benzene molecules. The former ones were modelled using SIMU restraints, the latter ones using ISOR restraints.

Alert level B

PLAT232_ALERT_2_B

Hirshfeld Test Diff (M-X) Nd2 --As3 . 13.0 s.u.

Author Response: Manual inspection of the structure model and the thermal ellipsoids did not indicate any problems. The alert is possibly caused by the high sd of the heavy atom positions.

[{K(18-crown-6)}$_2$(Cp''$_2$Nd)(μ_3-η^2:η^2:η^2-As$_7$)] (7)

Crystals of **7** exhibited a positional disorder of the 18-c-6 ligand and one TMS substituent. The former was modelled with *FragmentDB*,[10, 11] the latter using moderate to strong SIMU restraints. Additionally, compound **7** shows signs of twinning, leading to intense secondary q-peaks for all heavy atom positions.

Alert level A

PLAT971_ALERT_2_A

Check Calcd Resid. Dens. 1.21A From K1 4.38 eA-3

Check Calcd Resid. Dens. 0.93A From Nd1 3.68 eA-3

Author Response: The residual electron density is likely caused by untreated twinning. This leads to a second set of clearly visible q-Peaks for every heavy atom in the core structure. However, the chemical abundance of the twinned/disordered structure is too low, to be modelled satisfactorily.

Alert level B

PLAT910_ALERT_3_B

Missing # of FCF Reflection(s) Below Theta(Min). 11 Note

Author Response: The unit cell dimensions are large enough to reasonably explain missing low angle reflections to be caused by the beamstop.

PLAT971_ALERT_2_B

Check Calcd Resid. Dens. 0.86A From Nd1 2.56 eA-3

Check Calcd Resid. Dens. 1.01A From As1 2.51 eA-3

Author Response: The residual electron density is likely caused by untreated twinning. This leads to a second set of clearly visible q-Peaks for every heavy atom in the core structure. However, the chemical abundance of the twinned/disordered structure is too low, to be modelled satisfactorily.

Table S1: Crystal data and structure refinement of **1**, **2**, and **4** (La (**1**) and Ce (**2** and **4**) compounds).

**Compound**	**1**	**2**	**4**
**Formula**	$C_{83}H_{135}As_7KLa_2O_6Si_8$	$C_{77}H_{132}As_7Ce_2KO_6Si_8$	$C_{96}H_{164}As_{14}Ce_2K_2O_{12}Si_8$
**$D_{calc.}$/ g cm^{-3}**	1.483	1.495	1.648
**μ/mm^{-1}**	3.234	3.421	4.531
**Formula Weight**	2294.98	2222.32	3142.30
**Colour**	clear yellow	clear red	clear red
**Shape**	block	irregular	rod
**Size/mm^3**	0.37×0.31×0.23	0.31×0.22×0.17	0.27×0.17×0.10
**T/K**	100	100	100
**Crystal System**	triclinic	monoclinic	triclinic
**Space Group**	P-1	$P2_1/n$	P-1
**a/Å**	15.7346(5)	20.0117(4)	11.2230(14)
**b/Å**	18.2731(6)	18.0444(3)	16.579(2)
**c/Å**	19.7473(7)	27.5019(6)	18.693(3)
**α/°**	106.435(3)	-	72.888(10)
**β/°**	102.572(3)	96.273(2)	89.816(11)
**γ/°**	100.222(3)	-	73.008(10)
**V/Å^3**	5139.0(3)	9871.5(3)	3165.8(8)
**Z**	2	4	1
**Z'**	1	1	0.5
**Wavelength/ Å**	0.71073	0.71073	0.71073
**Radiation type**	Mo K_α	Mo K_α	Mo K_α
**Θ_{min}/°**	1.927	2.051	2.273
**Θ_{max}/°**	31.594	30.288	31.642
**Measured Refl's.**	55039	60541	28716
**Indep't Refl's**	27226	25742	16017
**Refl's I≥2 σ(I)**	19894	17981	11474
**R_{int}**	0.0401	0.0327	0.0372
**Parameters**	1051	977	869
**Restraints**	222	78	288
**Largest Peak**	4.346	2.261	1.098
**Deepest Hole**	-1.570	-0.621	-0.969
**GooF**	1.027	0.974	1.022
**wR_2 (all data)**	0.1760	0.0749	0.1279
**wR_2**	0.1616	0.0674	0.1138
**R_1 (all data)**	0.0880	0.0678	0.0759
**R_1**	0.0629	0.0364	0.0478

Table S2: Crystal data and structure refinement of **3**, **5**, **6**, and **7** (Nd compounds).

**Compound**	**3**	**5**	**6**	**7**
**Formula**	$C_{74}H_{138}As_{14}K_2Nd_2O_{12}Si_8$	$C_{77}H_{129}As_3KNd_2O_6Si_8$	$C_{80}H_{132}As_7KNd_2O_6Si_8$	$C_{58}H_{102}As_7K_2NdO_{12}Si_4$
$D_{calc.}$/ g cm^{-3}	1.773	1.270	1.504	1.557
μ/mm^{-1}	5.466	2.172	3.502	3.784
**Formula Weight**	2860.12	1927.85	2266.59	1850.63
**Colour**	clear yellow	clear red	clear yellow	clear orange
**Shape**	plate	fragment	block	fragment
**Size/mm³**	0.11×0.08×0.04	0.24×0.20×0.13	0.36×0.24×0.13	0.35×0.20×0.14
T/K	100	150	100	100
**Crystal System**	monoclinic	triclinic	triclinic	monoclinic
**Space Group**	$C2/c$	P-1	P-1	$P2_1/n$
a/Å	41.5910(16)	11.7764(13)	15.6899(7)	14.2709(2)
b/Å	13.2497(4)	17.4676(14)	18.4469(10)	31.9950(6)
c/Å	19.5306(8)	26.292(4)	20.1223(11)	17.4941(3)
α/°	-	84.855(9)	111.299(4)	-
β/°	95.516(3)	79.390(9)	104.839(4)	98.6780(10)
γ/°	-	71.581(8)	100.119(4)	-
**V/Å³**	10712.9(7)	5040.6(10)	5006.3(5)	7896.3(2)
Z	4	2	2	4
Z'	0.5	1	1	1
**Wavelength /Å**	0.71073	0.71073	0.71073	0.71073
**Radiation type**	Mo K_α	Mo K_α	Mo K_α	Mo K_α
Θ_{min}/°	2.095	1.438	1.980	2.355
Θ_{max}/°	30.324	27.228	31.667	29.104
**Measured Refl's.**	28105	47603	48707	57719
**Indep't Refl's**	13590	22194	26464	18080
**Refl's I≥2 σ(I)**	8536	16809	19574	15489
R_{int}	0.0492	0.0308	0.0493	0.0296
**Parameters**	688	1108	961	946
**Restraints**	378	156	0	936
**Largest Peak**	1.549	1.892	1.621	3.375
**Deepest Hole**	-0.932	-1.162	-1.762	-1.102
**GooF**	1.024	1.086	1.019	1.168
wR_2 **(all data)**	0.1332	0.1169	0.1648	0.1949
wR_2	0.1136	0.1053	0.1512	0.1898
R_1 **(all data)**	0.1043	0.0735	0.0847	0.0873
R_1	0.0535	0.0470	0.0600	0.0769

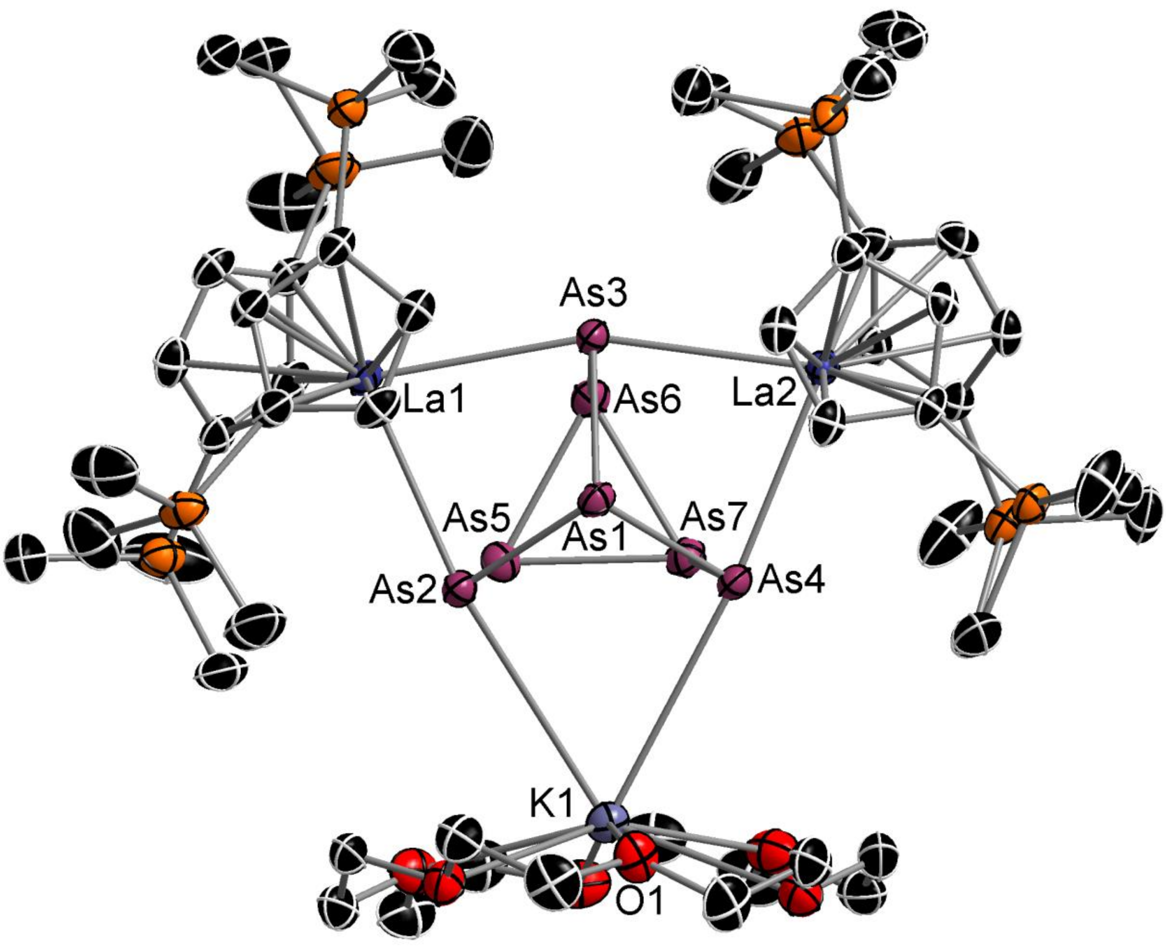

Figure S8: Molecular structure of **1** in the solid state displayed with an ellipsoid probability of 50 %. Hydrogen atoms and solvent molecules are omitted for clarity. Selected bond lengths [Å] and angles [°]: La1-As2 3.0699(7), La1-As3 3.0699(7), La2-As3 3.0803(6), La2-As4 3.0751(7), K-As2 3.6863(14), K-As4 3.621(2), As1-As2 2.4023(8), As1-As3 2.4714(8), As1-As4 2.4079(8), As2-As5 2.3827(9), As3-As6 2.3968(9), As4-As7 2.3887(9), As5-As6 2.4795(9), As5-As7 2.4604(9), As6-As7 2.4625(9), As2-La1-As3 76.08(2), As3-La2-As4 75.36(2), As2-K-As4 59.98(2), As3-As1-As4 100.91(3), As2-As1-As4 98.83(3), As2-As1-As3 101.85(3), As1-As2-As5 99.79(3), As5-As6-As7 59.71(3), As6-As5-As7 59.80(3), As2-As5-As7 104.67(3), As2-As5-As6 106.67(3).

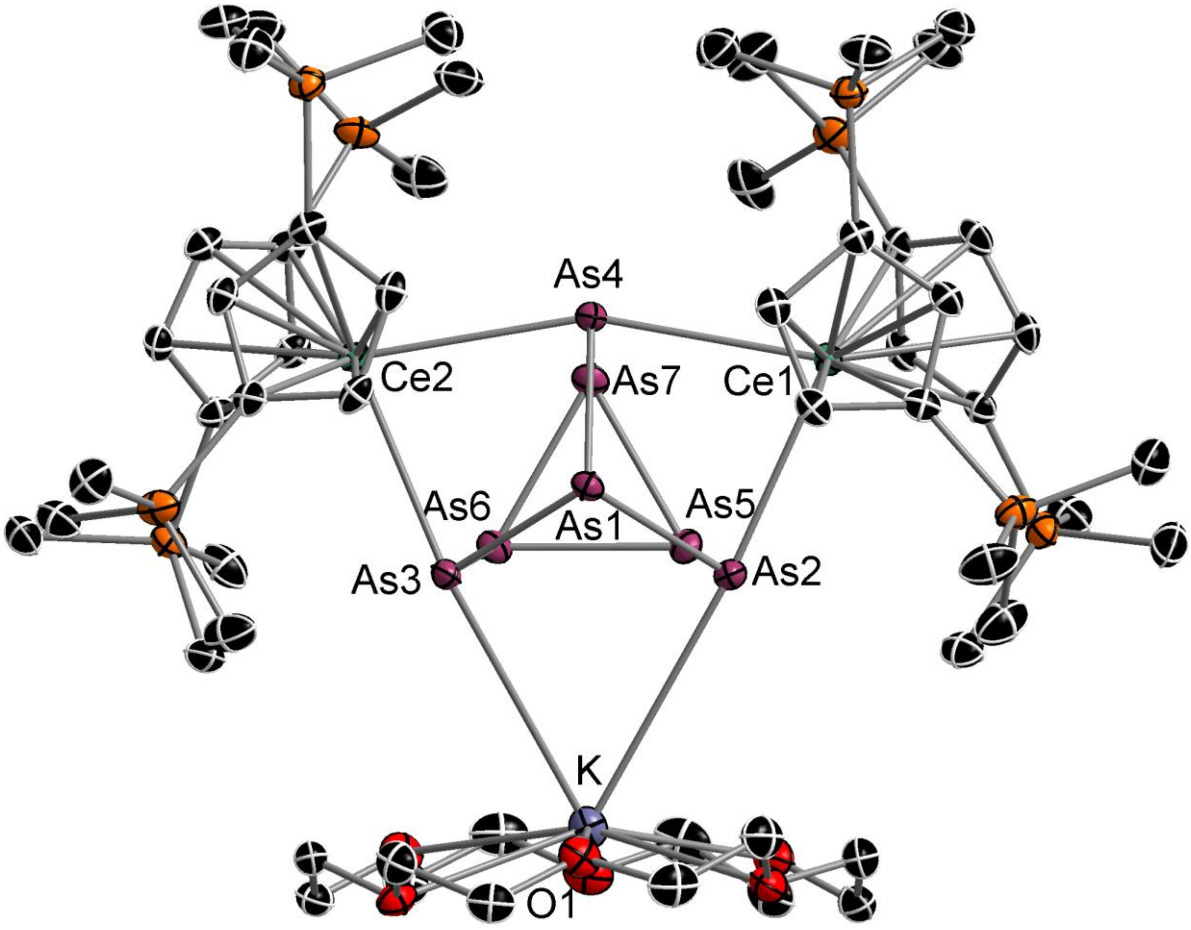

Figure S9: Molecular structure of **2** in the solid state displayed with an ellipsoid probability of 50 %. Hydrogen atoms and solvent molecules are omitted for clarity. Selected bond lengths [Å] and angles [°]: Ce1-As2 3.0460(4), Ce1-As4 3.1053(4), Ce2-As3 3.0412(4), Ce2-As4 3.0897(4), K-As2 3.7609(8), K-As3 3.7624(9), As1-As2 2.4101(5), As1-As3 2.4129(5), As1-As4 2.4777(5), As2-As5 2.3877(5), As3-As6 2.3845(5), As4-As7 2.4010(5), As5-As6 2.4654(5), As5-As7 2.4717(5), As6-As7 2.4805(5), As2-Ce1-As4 75.684(10), As3-Ce2-As4 76.752(10), As2-K-As3 58.230(14), As3-As1-As4 102.20(2), As2-As1-As4 101.06(2), As2-As1-As3 98.72(2), As1-As2-As5 99.82(2), As5-As6-As7 59.967(14), As6-As5-As7 60.318(14), As2-As5-As7 106.40(2), As2-As5-As6 104.07(2).

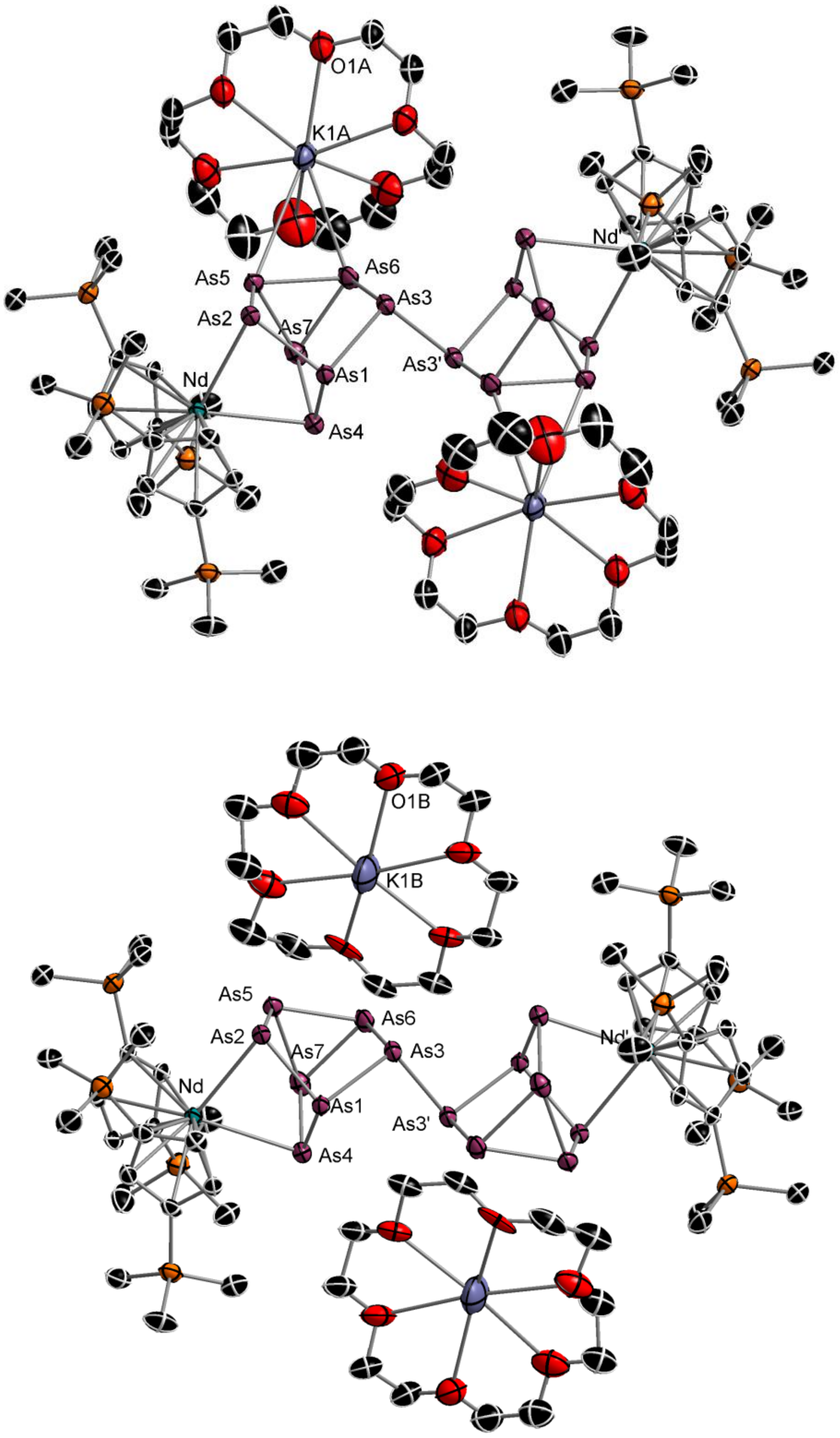

Figure S10: Molecular structure of **3** in the solid state displayed with an ellipsoid probability of 50 %. Hydrogen atoms and solvent molecules are omitted for clarity. Positional disorder of [K(18-crown-6)]$^+$ over two parts (0.75:0.25). Selected bond lengths [Å] and angles [°]**:** Nd-As2 3.0506(7), Nd-As4 3.0673(7), K1A-As5 3.956(3), K1A-As6 3.537(3), As1-As2 2.4186(9), As1-As3 2.4346(9), As1-As4 2.4010(10), As2-As5 2.4066(10), As3-As6 2.4098(10), As4-As7 2.3793(10), As5-As6 2.4620(10), As5-As7 2.4580(11), As6-As7 2.4516(10), As3-As3' 2.4522(14), As4-Nd-As2 76.85(2), As5-K-As6 37.84(4), As2-As1-As3 92.78(3), As4-As1-As3 103.35(3), As4-As1-As2 104.18(3), As1-As4-As7 99.29(3), As6-As5-As7 59.78(3), As5-As6-As7 60.03(3), As4-As7-As6 106.87(3), As4-As7-As5 106.88(4).

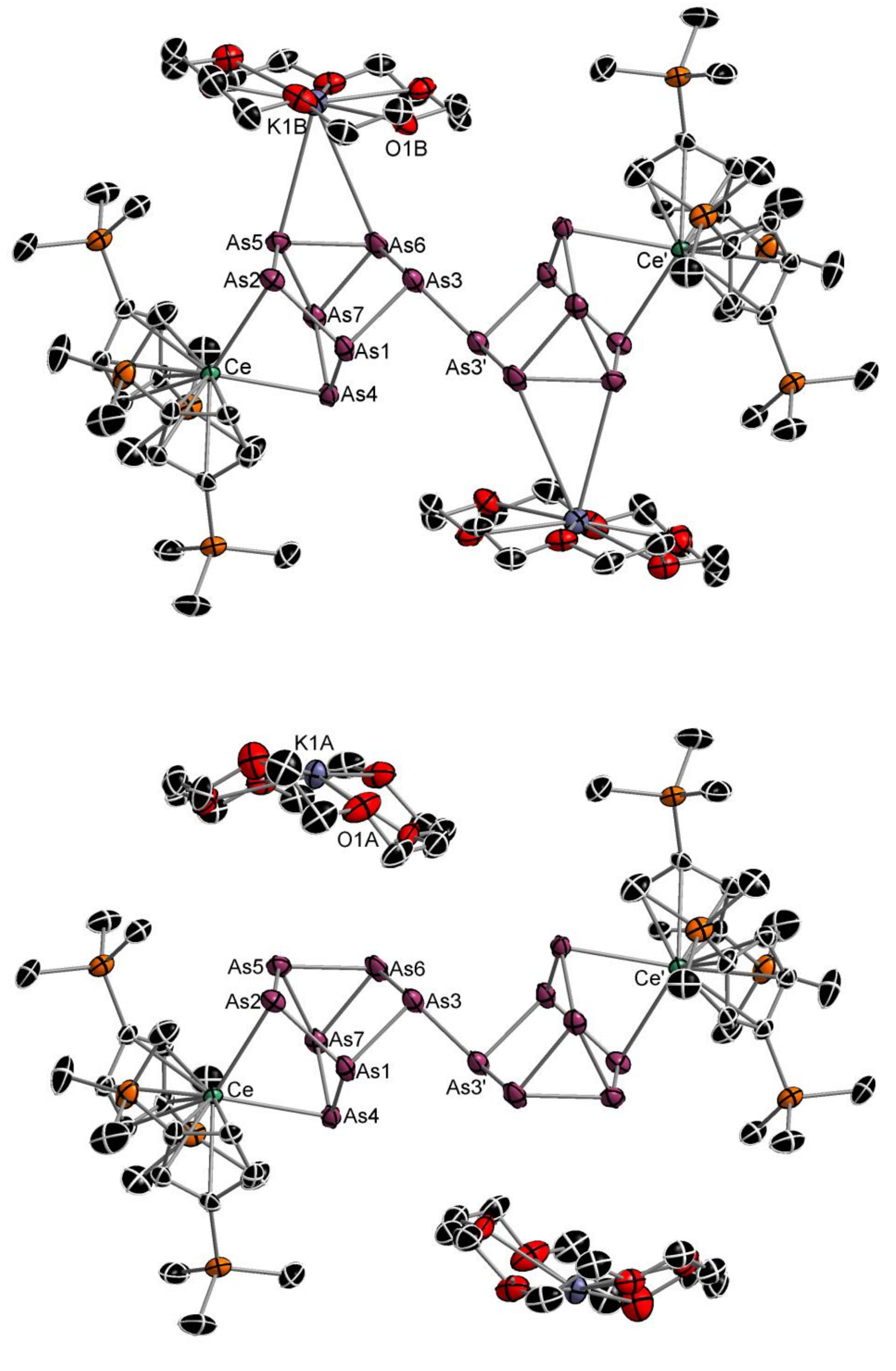

Figure S11: Molecular structure of **4** in the solid state displayed with an ellipsoid probability of 50 %. Hydrogen atoms and solvent molecules are omitted for clarity. Positional disorder of $[K(18\text{-crown-}6)]^+$ over two parts (0.5:0.5). Selected bond lengths [Å] and angles [°]**:** Ce-As2 3.0598(8), Ce-As4 3.0417(7), K1B-As5 3.650(3), K1B-As6 4.089(2), As1-As2 2.4220(8), As1-As3 2.4163(8), As1-As4 2.4100(9), As2-As5 2.4019(9), As3-As6 2.4215(9), As4-As7 2.3722(8), As5-As6 2.4565(9), As5-As7 2.4661(9), As6-As7 2.4490(10), As3-As3' 2.4468(13), As2-Ce-As4 77.84(2), As5-K-As6 36.45(3), As3-As1-As4 103.01(3), As2-As1-As4 104.98(3), As2-As1-As3 92.49(3), As1-As2-As5 99.25(3), As5-As6-As7 60.36(2), As6-As5-As7 59.67(3), As2-As5-As7 106.00(3), As2-As5-As6 105.41(3).

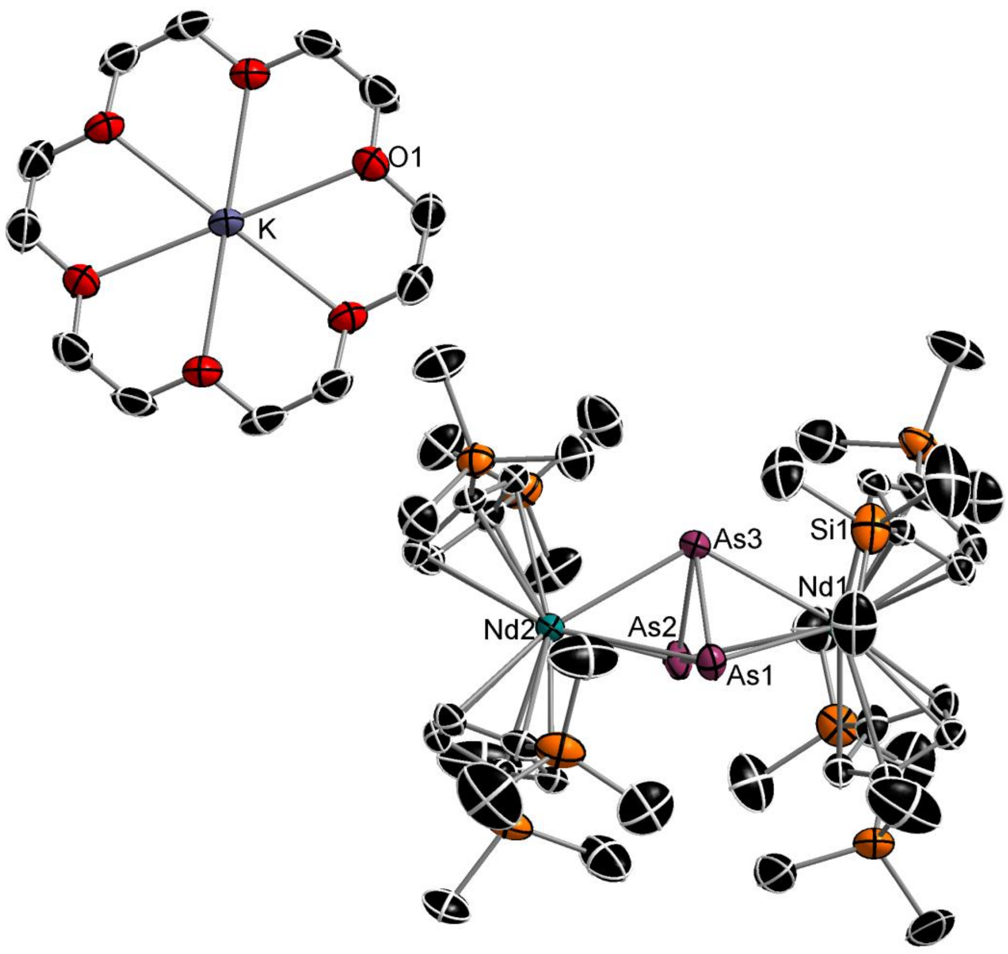

Figure S12: Molecular structure of **5** in the solid state displayed with an ellipsoid probability of 50 %. Hydrogen atoms and solvent molecules are omitted for clarity. Selected bond lengths [Å] and angles [°]**:** Nd1-As1 2.9367(7), Nd1-As2 3.0481(7), Nd1-As3 3.0232(7), Nd2-As1 3.0293(7), Nd2-As2 2.9311(7), Nd2-As3 3.0411(7), As1-As2 2.4388(8), As1-As3 2.4198(7), As2-As3 2.4228(8), As1-As2-As3 59.70(2), As1-As3-As2 60.48(2), As2-As1-As3 59.82(2).

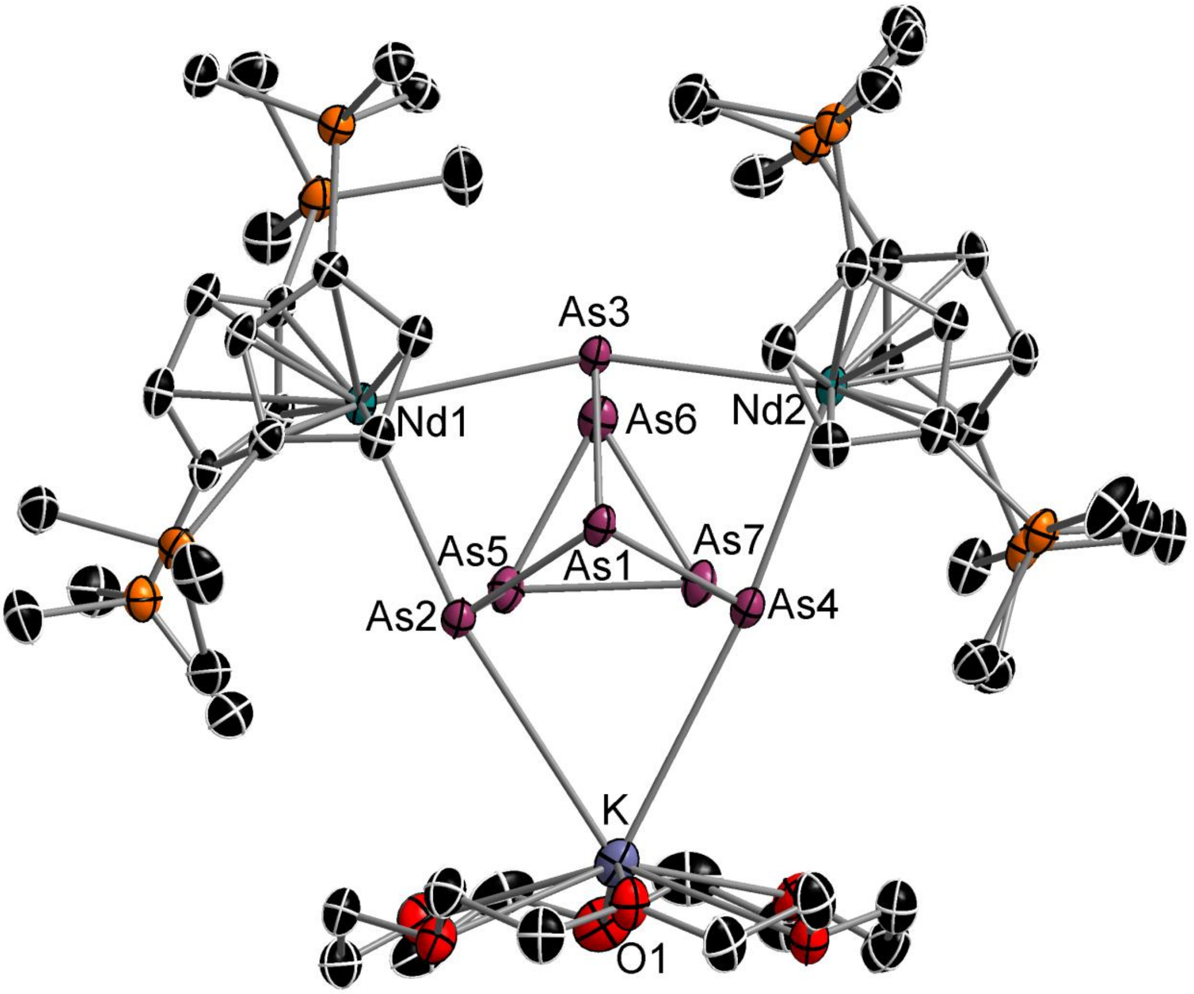

Figure S13: Molecular structure of **6** in the solid state displayed with an ellipsoid probability of 50 %. Hydrogen atoms and solvent molecules are omitted for clarity. Selected bond lengths [Å] and angles [°]**:** Nd1-As2 2.9974(7), Nd1-As3 3.0436(6), Nd2-As3 3.0390(6), Nd2-As4 3.0103(7), K-As2 3.710(2), K-As4 3.655(2), As1-As2 2.4055(9), As1-As3 2.4574(8), As1-As4 2.4026(8), As2-As5 2.3958(9), As3-As6 2.4056(9), As4-As7 2.3959(9), As5-As6 2.4646(10), As5-As7 2.4526(8), As6-As7 2.4516(9), As2-Nd1-As3 77.08(2), As3-Nd2-As4 76.539(17), As2-K-As4 59.92(3), As3-As1-As4 100.87(3), As2-As1-As4 99.84(3), As2-As1-As3 101.44(3), As1-As2-As5 99.63(3), As5-As6-As7 59.85(3), As6-As5-As7 59.81(3), As2-As5-As7 104.69(3), As2-As5-As6 106.18(3).

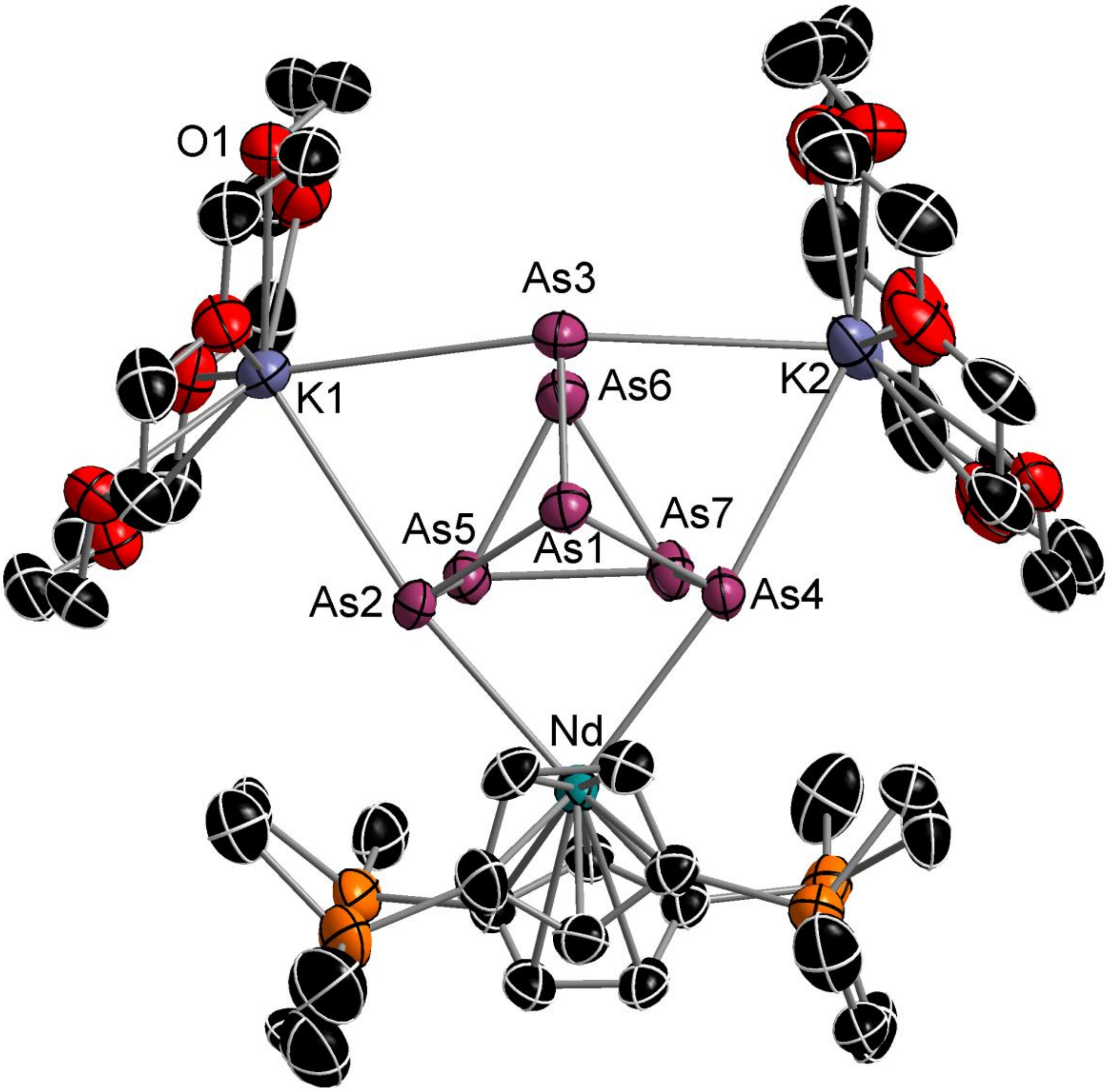

Figure S14: Molecular structure of **7** in the solid state displayed with an ellipsoid probability of 50 %. Hydrogen atoms and solvent molecules are omitted for clarity. Selected bond lengths [Å] and angles [°]**:** Nd-As2 2.9931(9), Nd-As4 2.9775(9), K1-As2 3.422(2), K1-As3 3.686(2), K2-As3 3.674(2), K2-As4 3.499(2), As1-As2 2.4349(12), As1-As3 2.3790(13), As1-As4 2.4376(11), As2-As5 2.3879(13), As3-As6 2.3537(14), As4-As7 2.3849(12), As5-As6 2.4851(13), As5-As7 2.4708(11), As6-As7 2.4904(13), As2-Nd-As4 78.23(2), As2-K1-As3 63.13(4), As3-K2-As4 62.49(4), As3-As1-As4 101.26(4), As2-As1-As4 101.26(4), As2-As1-As3 101.48(4), As1-As2-As5 98.85(4), As5-As6-As7 59.55(3), As6-As5-As7 60.33(4), As2-As5-As7 105.39(4), As2-As5-As6 104.13(4).

References

1. C. Schoo, S. Bestgen, A. Egeberg, J. Seibert, S. N. Konchenko, C. Feldmann and P. W. Roesky, *Angew. Chem. Int. Ed.*, 2019, **58**, 4386-4389.
2. M. C. Cassani, D. J. Duncalf and M. F. Lappert, *J. Am. Chem. Soc.*, 1998, **120**, 12958-12959.
3. C. T. Palumbo, L. E. Darago, M. T. Dumas, J. W. Ziller, J. R. Long and W. J. Evans, *Organometallics*, 2018, **37**, 3322-3331.
4. N. Reinfandt, N. Michenfelder, C. Schoo, R. Yadav, S. Reichl, S. N. Konchenko, A. N. Unterreiner, M. Scheer and P. W. Roesky, *Chem. Eur. J.*, 2021, **27**, 7862-7871.
5. D. F. Evans, *J. Chem. Soc.*, 1959, 2003-2005.
6. G. Sheldrick, *Acta Crystallogr. Sect. A*, 2008, **64**, 112-122.
7. G. Sheldrick, *Acta Crystallogr. Sect. C*, 2015, **71**, 3-8.
8. G. Sheldrick, *Acta Crystallogr. Sect. A*, 2015, **71**, 3-8.
9. O. V. Dolomanov, L. J. Bourhis, R. J. Gildea, J. A. K. Howard and H. Puschmann, *J. Appl. Crystallogr.*, 2009, **42**, 339-341.
10. D. Kratzert, J. J. Holstein and I. Krossing, *J. Appl. Crystallogr.*, 2015, **48**, 933-938.
11. D. Kratzert and I. Krossing, *J. Appl. Crystallogr.*, 2018, **51**, 928-934.
